# Supplementary material for: Intra-ripple frequency accommodation in an inhibitory network model for hippocampal ripple oscillations
Source: PLoS Comput Biol. 2024 Feb 20;20(2):e1011886. doi: 10.1371/journal.pcbi.1011886 (PMC10923461; doi:10.1371/journal.pcbi.1011886)
Supplement: S1 Appendix — The Gaussian-drift approximation under constant drive is compared to spiking network simulations for a range of noise intensities D and inhibitory coupling strengths K (Fig A). The parameter dependencies of the point of full synchrony are captured well by the theory (Fig B). (PDF) [file pcbi.1011886.s001.pdf]

**S1 Appendix.**

**Performance evaluation of the Gaussian-drift approximation for constant drive**

We confirmed with numerical simulations that for strong enough drive the Gaussian-drift approximation works for a wide parameter regime. Our spiking network model has only four parameters: the noise intensity $D$, the inhibitory coupling strength $K$, the synaptic delay $\Delta$, and the reset potential $V_R$ (see Eq (3)). Since time can be rescaled to units of the membrane time constant, we do not count the membrane time constant as an independent parameter. We show here a two-dimensional parameter exploration covarying the noise intensity $D$ and the inhibitory coupling strength $K$ (Fig A). Subsequently we will briefly comment on the role of the synaptic delay and the reset potential. To quantify the performance of the Gaussian-drift approximation, we introduce a performance index that takes into account the error in the estimate of the network frequency and the proportion of the relevant range of external drives (from the Hopf bifurcation up to the point of full synchrony) that is covered by the theory (Methods, Eq (55)).

We find that performance is good for a wide range of parameters (Fig Ab). As expected from a drift-based approximation, the performance decreases for larger noise intensity (Fig A, large $\sqrt{D}$). At high noise and weak coupling the range of external drives, for which the theory applies, decreases markedly. The wider the Gaussian density and the weaker the inhibitory feedback, the harder it is to satisfy our requirement (b) of the bulk of the membrane potential density being pushed subthreshold in between population spikes (Fig Ad). In the extreme case of $\sqrt{D} = 0.4,\ K = 2$ this criterion is never fulfilled (Fig Ab–d, bottom right).

We also observe a dip in performance for low noise and low coupling strength (Fig Ab–d, bottom left). This, however, does not reflect a shortcoming of our approximation but rather a departure of the spiking network dynamics from the regime of interest: If noise and coupling strength are very low relative to the distance between threshold and reset, the fire-and-reset mechanism introduces a permanent multimodality in the membrane potential distribution. Units that have spiked in one cycle are less likely to spike in the next. Such a *clustered* activity (see also [1]) does not correspond to our regime of interest and can of course not be captured by our approximation. To get back to the regime of interest for the given level of low noise and low coupling, one could increase the reset potential, essentially introducing a third dimension to our parameter exploration.

Our parameter exploration reveals that the network frequency beyond the Hopf bifurcation depends strongly on the inhibitory coupling strength (Fig Ac, variation along vertical axis $K$). Previous studies focusing on linear stability analysis around the Hopf bifurcation [2,3] have suggested that the network frequency is set primarily by the (fast) synaptic time constants and depends only weakly on other parameters, which our simulations confirm (see Fig Ac, red markers). Here we see, however, that further away from the bifurcation other parameter dependencies *do* play a role. For strong coupling ($K = 50$, Fig Ac, top row) we even observe a drop in the network frequencies to slow gamma range. Our approximation captures this dependency very well.

The point of full synchrony is predicted well (Fig Ac). Its parameter dependencies are illustrated in Fig B: while *quantitatively* the theoretical estimate produces an error that becomes larger for stronger noise (Fig B, *error*), the *qualitative* dependency of the point of full synchrony on the noise and coupling strength is captured well (Fig B, *theory* vs *simulation*). The theory predicts that stronger external drive is required to achieve full synchrony, if the noise is stronger, which is confirmed in simulations (Fig B bottom). A similar, albeit weaker, dependence can be found for the inhibitory coupling strength: If the network is coupled more strongly, stronger external drive is required to achieve full synchrony.

What about the remaining two parameters, $\Delta$ and $V_R$? We have covaried the synaptic delay in a biologically plausible range ($\Delta \in [0.5, 2]$ ms) with either the noise intensity or the coupling strength (not shown here). The performance of the Gaussian-drift approximation is largely unaffected by the synaptic delay, which merely shifts the overall network frequencies to higher or lower values as predicted by [2]. As mentioned before, the reset potential $V_R$ can introduce permanent multimodality in the distribution of membrane potentials if it is far from threshold relative to noise intensity and coupling strength. As long as approximate unimodality is ensured, the reset potential does not influence the performance of the approximation much.

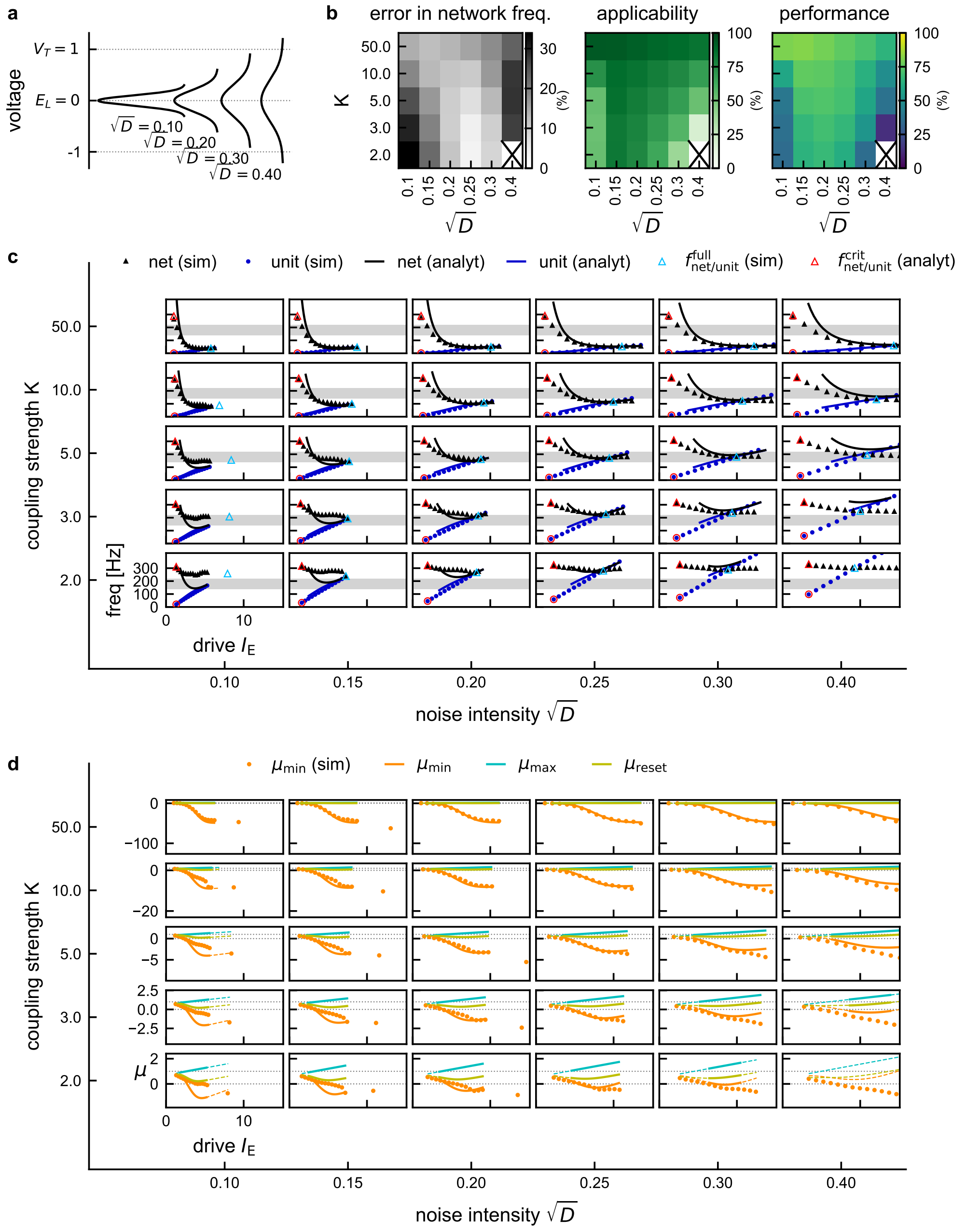


**Fig A. Performance of the Gaussian-drift approximation.** Noise intensity $D$ and coupling strength $K$ were covaried in the range $\sqrt{D} \in [0.1, 0.4]$ and $K \in [2, 50]$. (a) Visualization of the width of the Gaussian voltage density $p(V, t)$ for different noise intensities $D$. (b) performance index reveals optimal parameter regime in terms of approximation error and applicability of the theory. (c) comparison of network frequencies (black) and unit firing rates (blue) in theory (line) and simulation (markers). Red markers: Hopf bifurcation. Blue triangle: point of full synchrony in spiking network simulation. All theory curves are shown for the respective range $[I_E^{min}, I_E^{max}]$ (see Methods, Eq (52)). (d) local extrema $\mu_{max}, \mu_{min}$ and reset $\mu_{reset}$ of the mean membrane potential. Dashed colored lines: theory does not apply. Full colored lines: theory applies, $I_E \in [I_E^{min}, I_E^{full}]$. Note that $\mu_{min}$ is a quasi monotonically decaying function of the drive, except for very weak coupling.

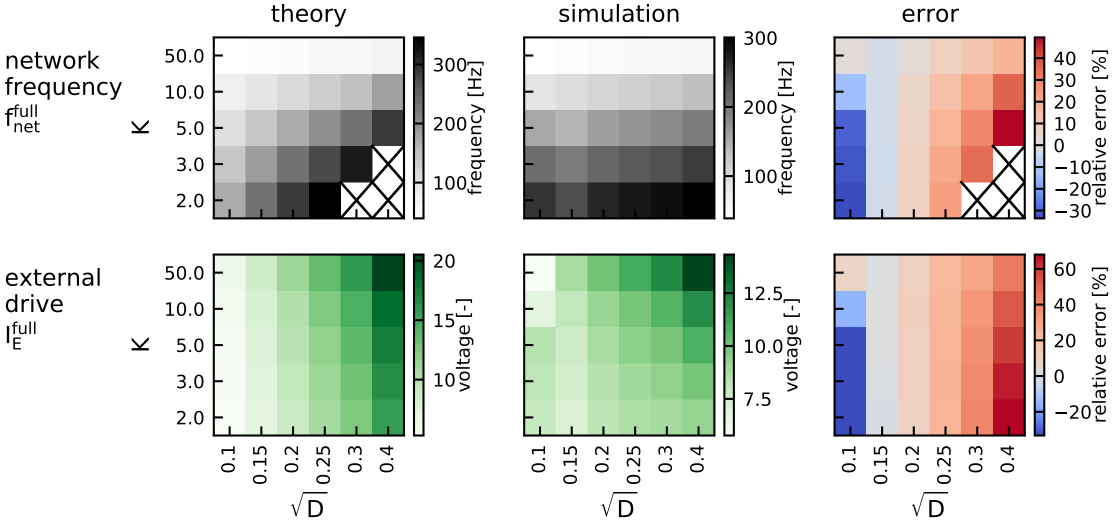


**Fig B. Parameter dependencies of the point of full synchrony.** External drive $I_E^{full}$ at which full synchrony is reached (bottom) and the corresponding network frequency $f_{net}^{full}$ (top), as predicted by theory (left, Eq (51)) vs. as observed in spiking network simulation (middle). The right panel shows the relative deviation of the theoretical prediction from the simulation result. Same parameter exploration as in Fig A. Crosses mark the parameter settings for which the point of full synchrony lies outside the regime of applicability of our theory ($I_E^{full} > I_E^{max}$, cf. Fig Ad).

## References


1. Brunel N, Hansel D. How Noise Affects the Synchronization Properties of Recurrent Networks of Inhibitory Neurons. Neural Comput. 2006;18(5):1066–1110. doi:10.1162/neco.2006.18.5.1066.

2. Brunel N, Hakim V. Fast Global Oscillations in Networks of Integrate-and-Fire Neurons with Low Firing Rates. Neural Comput. 1999;11(7):1621–1671. doi:10.1162/089976699300016179.

3. Brunel N, Wang XJ. What determines the frequency of fast network oscillations with irregular neural discharges? I. Synaptic dynamics and excitation-inhibition balance. J Neurophysiol. 2003;90(1):415–430. doi:10.1152/jn.01095.2002.
