# Supplementary material for: Intra-ripple frequency accommodation in an inhibitory network model for hippocampal ripple oscillations
Source: PLoS Comput Biol. 2024 Feb 20;20(2):e1011886. doi: 10.1371/journal.pcbi.1011886 (PMC10923461; doi:10.1371/journal.pcbi.1011886)
Supplement: S2 Appendix — A covariation of network architecture and stimulus profile demonstrates that IFA is modulated by, but occurs largely independent of, the shape of the asymptotic network frequency as a function of the external drive (Fig A). Furthermore, we illustrate that a simple square pulse cannot elicit IFA in the feedback-based inhibition-first model. (PDF) [file pcbi.1011886.s002.pdf]

S2 Appendix.

The influence of network architecture and the shape of the external drive on the asymptotic and instantaneous ripple oscillation dynamics.

Fig A, panel 1 illustrates that the shape of the asymptotic network frequency as a function of the external drive varies with network architecture and can be any of the following: decrease followed by increase (a1, c1), monotonic increase (b1), monotonic decrease (d1). The qualitative mechanism of IFA, however, is preserved (Fig A, panels 2–4): instantaneous network frequencies are above the asymptotic frequencies while the drive increases (most red dots are above the black curve), and below while the drive decreases (most blue dots are below the black curve). Slight deviations from this rule (blue dots in a2) can be attributed to the finite size of the network and the somewhat arbitrary choice of the reference drive within a given ripple cycle. The shape of the instantaneous frequency response to SPW-like drive depends both on the shape of the asymptotic network frequencies, which varies across network architectures (panel 1), as well as the input profile (panels 2-4). Except for b4, all transient double ramp simulations exhibit IFA ($\chi_{\mathrm{IFA}} < 0$ in panels 2–4). Due to the monotonically decreasing shape of the asymptotic frequency in our reduced model (d1), the strongest IFA is achieved with a right-shifted drive (d4). In the more biologically plausible network architectures (a–c) the asymptotic frequency depends more weakly on the drive or rises proportionally with it (a1–c1). In these cases the strongest IFA is achieved with a left-shifted drive (a3–c3).

When stimulated with a square pulse (panel 5) none of the networks exhibit significant IFA ($\chi_{\mathrm{IFA}} \sim 0$ in a5–d5). In that sense a double ramp drive can be considered the simplest form of SPW-like drive that can elicit IFA in the feedback-based inhibition-first model.

Fig A. Asymptotic and instantaneous network frequencies depending on network architecture and stimulus profile.

The shape of the asymptotic network frequencies under constant drive (top, 1), and the instantaneous network frequencies under SPW-like drive (bottom, 2–5) compared across four different network architectures: (a) Original network from Donoso et al. [1] (c.f. their Fig. 1D): Conductance-based inhibitory coupling with double-exponential filter, excitatory Poisson spiking input from partially overlapping presynaptic cell populations. (b) Donoso et al. network [1] with Poisson spiking input replaced by i.i.d. Gaussian white noise. Noise intensity grows as the square root of the mean drive (Poisson-like). All parameters as in (a). (c) Reduced model presented in this manuscript (Eq (20)) with an absolute refractory period $\tau_{\mathrm{ref}} = 1$ ms, and increased noise intensity $D = 0.2$ and synaptic delay $\Delta = 1.5$ ms to ensure approximate unimodality of the membrane potential distribution. All other parameters as in Table 2. (d) Reduced model with default parameters (Table 2, no refractory period). For comparability all simulations were performed at a fixed network size of $N = 200$.

Panel 1 shows the asymptotic network frequency (black) and mean unit firing rate (blue) for a range of (constant) external drives. The ripple band is marked in gray. Panels 2–5 show the instantaneous network frequencies (colored/gray dots) in response to SPW-like drive (gray line). In panels 2–4 the SPW-like drive is modelled as a double ramp up to the approximate point of full synchrony of the respective network (vertical dotted lines in panel 1). The shape of the double ramp is varied between symmetric (2), left-shifted (3), or right-shifted (4). In panel 5 the networks are stimulated with a square pulse of amplitude $0.8 I_{\mathrm{E}}^{\mathrm{full}}$.

The instantaneous frequencies in response to symmetric drive (2) are colored according to the time of their measurement (red to blue as time increases in panel 2). The same instantaneous frequency estimates are plotted again in panel 1, now against the momentary drive at the time of their measurement. Vice versa, the asymptotic frequencies shown in panel 1 are interpolated and replotted in panels 2–5 depending on the momentary drive (black line). IFA is quantified by the slope χ_{IFA} of a linear regression over the instantaneous frequencies (thin gray line).

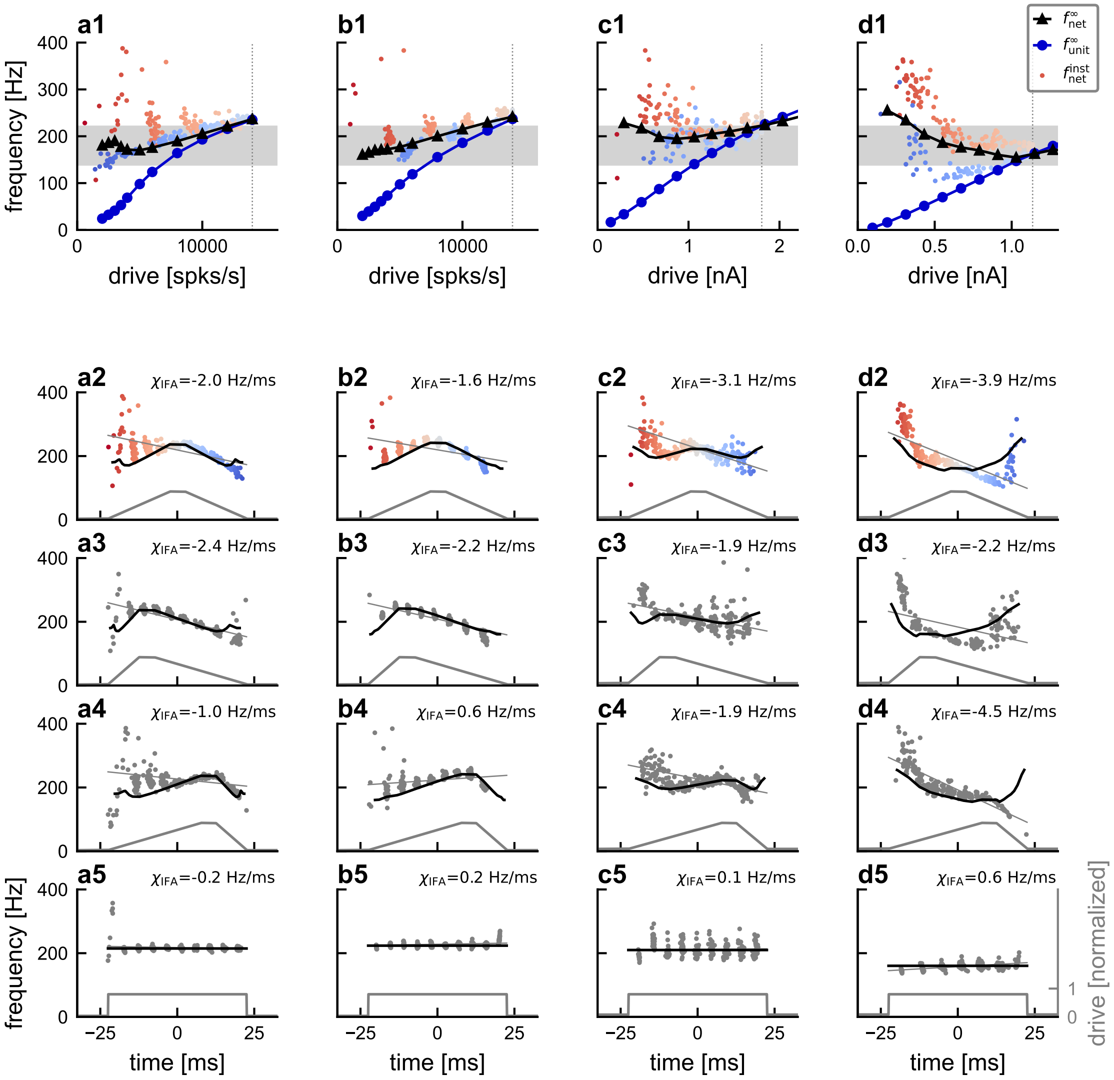

Fig A. Asymptotic and instantaneous network frequencies depending on network architecture and stimulus profile.
Caption on previous page.

References

1. Donoso JR, Schmitz D, Maier N, Kempter R. Hippocampal ripple oscillations and inhibition-first network models: Frequency dynamics and response to GABA modulators. J Neurosci. 2018;38(12):3124–3146. doi:10.1523/JNEUROSCI.0188-17.2018.
