# Supplementary figures and images for: Intra-ripple frequency accommodation in an inhibitory network model for hippocampal ripple oscillations
Source: PLoS Comput Biol. 2024 Feb 20;20(2):e1011886. doi: 10.1371/journal.pcbi.1011886 (PMC10923461; doi:10.1371/journal.pcbi.1011886)

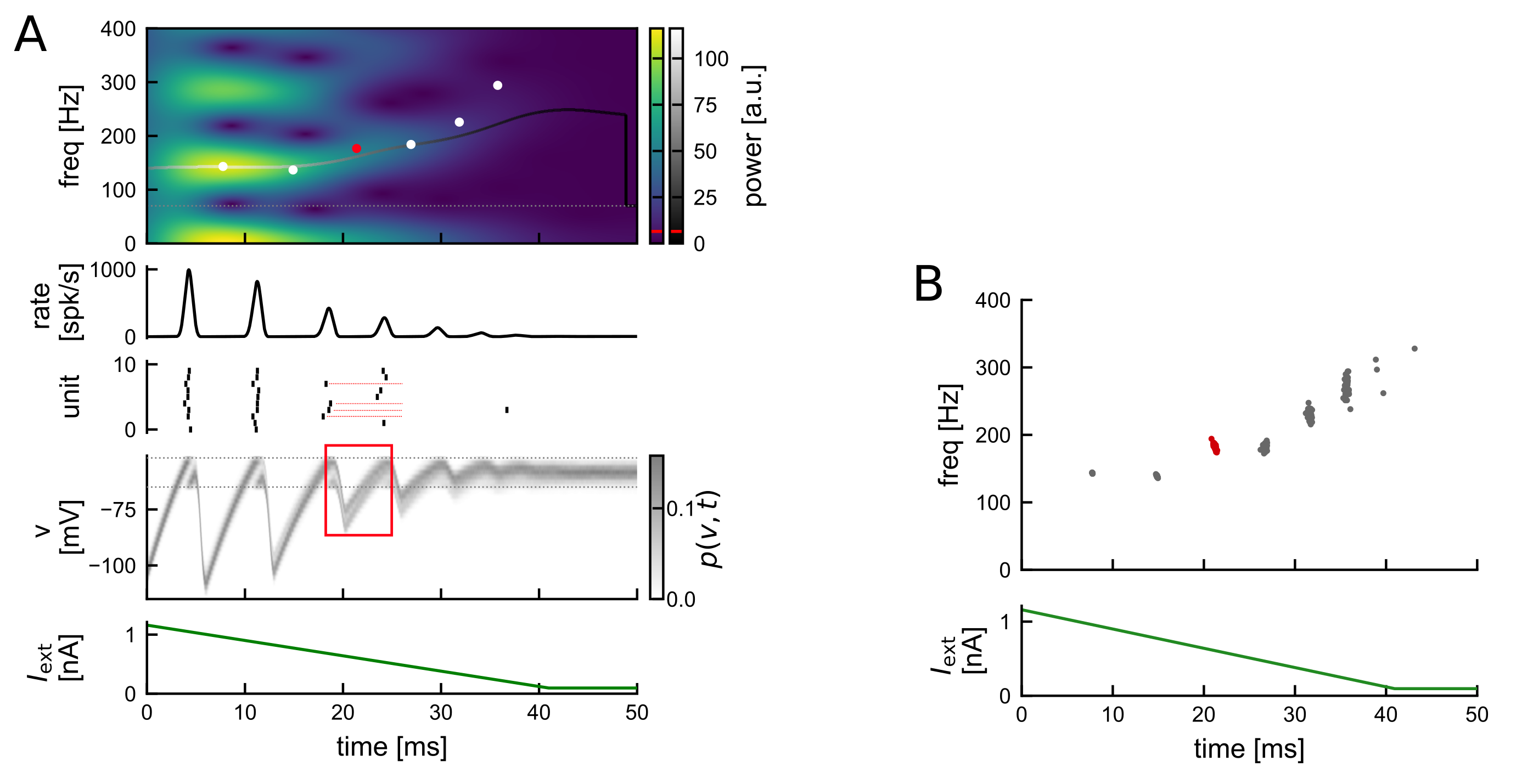

Supplement: S1 Fig — (A) Same layout as in Fig 2A: Spiking network response to an isolated downwards ramp stimulus with the same slope as in Fig 2D, middle, after time t > 10 ms (N = 10, 000). Note that units that participate in the third population spike tend not to spike in the fourth population spike (red lines in raster plot), which is an indication of a residual bimodality in the membrane potential distribution from one cycle to the next (only faintly visible in voltage plot, see red square). (B) Same layout as Fig 2B: instantaneous network frequencies pooled together from 50 such ramp-down-only simulations. What appears as a continuous non-monotonic “wiggle” in the instantaneous frequencies of Fig 2D, middle (gray dots) is now clearly identifiable as a single outlier cycle (marked in red). (TIF) [file pcbi.1011886.s003.tif]
