# Supplementary material for: Intra-ripple frequency accommodation in an inhibitory network model for hippocampal ripple oscillations
Source: PLoS Comput Biol. 2024 Feb 20;20(2):e1011886. doi: 10.1371/journal.pcbi.1011886 (PMC10923461; doi:10.1371/journal.pcbi.1011886)
Supplement: Attachment — Submitted filename: Review_fnl.pdf [file pcbi.1011886.s004.pdf]

Reviewer's report for PLoS CB on
"Intra-ripple frequency accommodation in an inhibitory network model for hippocampal ripple oscillations"
by Natalie Schieferstein, Tilo Schwalger, Benjamin Lindner, Richard Kempter

Overview.
This paper reports on the modeling and analysis of IFA. It provides a follow-up to an earlier paper from the Kempter lab: Donoso, Schmitz, Maier, Kempter (2018) J Neurosci

The spiking model involves all-to-all coupled LIF units; coupling is by an inhibitory current pulse with propagation delay of 1.2 ms. The application is SWRs in CA1 driven by excitatory drive I_E from CA3; I_E is represented by a linear ramp-up then plateau then linear ramp-down with total duration of 10s ms. The goal is to provide and to analyze a MF-like approximation for the experimental and simulated observations: generally, the intra-SWR cycle frequency shows 'accommodation', decreasing throughout the stimulus but with more or less increasing frequency during the late down-ramp, depending on the speed of the down-ramp.

The treatment proceeds in a well-organized hierarchy to describe the response to increasingly detailed features of the transient input. The authors begin with strictly steady input and approximating the steady response in terms of network frequency and cell frequency vs I_E (Fig 4). This analytic part alone is quite nice and of value. Then, they provide math analysis of the ramping up and ramping down in stepwise fashion, with an I_E incremental step occurring at the end of each response cycle; finally they mathematically treat the linear ramp case.

The response during ramping deviates from close tracking of the asymptotic frequency, depending on the speed and direction of the changing I_E. There is asymmetry (hysteresis) between increasing and decreasing I_E. A substantial portion of the paper involves accounting for the deviations with simulation and math analysis; an attempt with various approximations to describe/dissect the response to transient stimuli.

The math analysis is insightful with clever approximations/assumptions, based on a Fokker-Planck description of the noisy response of the LIF network. The authors provide a valuable and novel analytic treatment for responses to a transient input. But some readers may prefer a more direct demonstration of the gaussian approximation without the detailed analysis (see our suggestion below, just before Major Concerns).

The paper is well-organized and clearly written, with generally understandable figures and captions. It's long -- with Methods being longer than the main text (20 pages compared to 19 pages) and 4 pages of Supp Info.

Curiously, the authors' I-only model shows decreasing asymptotic network frequency with increasing I_E. This behavior contrasts with the network frequency vs I_E relation seen in many cell-based models for an I-only network, including that in Donoso et al. (2018), Fig 1D, and, for example, in models for ING-type gamma oscillations the frequency typically increases with I_E (Fig 12A in Wang & Buzsáki 1996; Fig 4C in Bartos, Vida & Jonas 2007).

We offer the following suggestion before listing our major/minor concerns.
The approximations, simplifying assumptions, and analysis are impressive for arriving at an analytical solution. A key factor is the gaussian profile assumption and by-passing of the cellular-threshold resetting. The DDE by itself is a valuable reduction and can be integrated numerically for demonstrating key features of IFA. Figures 3 and 4 are quite good for the case of steady input drive. Eventually, after going through much analysis in the paper, I realized that **simulations of** IFA **for the direct numerical**

integration of the DDE (as in Fig 6) could be shown earlier, say, just after Fig 4. I suggest a comparison of the DDE simulations with the LIF network simulations to directly illustrate IFA (by simulation for a piecewise linear $I_E(t)$) with time courses like those in, say, Figs 2A, 3A, 3B. Many readers may be satisfied with these demonstrations and the text could offer them the option to by-pass the results and details of the analytical approach (pages 12-16 and in Methods, pages 27-33). You could include the time course of the mean of V across cells from the spiking network, likewise for Fig 1. The other figures could remain pretty much as they are.

Major Concerns:

1. In this LIF I-only network model the synaptic current I_{syn} is a delta-pulse, decreasing the post-synaptic V(t) by J/N=65 mV/10000. There is not a finite time course or decay time constant for I_{syn}. This representation leads to stronger post-synaptic hyperpolarization (decreased μ_{min}) with increased I_E. The hyperpolarization can be 2-3 x 13 mV below V_{rest} (see Fig 2A), say to -100 mV or more negative. I urge you to make this point very explicitly in the text. Please provide intuitive explanation for this decrease of μ_{min} with increasing I_E. Is it that, larger I_E tends to speed the passage through threshold, enabling more spikes to occur in a firing episode (greater saturation, larger value of s) and thereby more inhibitory current pulses, decreased μ_{min}? Is this the basis for decreased asymptotic frequency with I_E? Please give the reader an intuitive explanation for the decrease of μ_{min} with I_E.
2. With conductance-based inhibitory synaptic current there could be saturation of the synaptic activation variable, w(t), according to $I_{syn} = G_{syn}\, w(t)\, (V-V_{syn})$ (with modestly hyperpolarizing V_{syn} and $0 \leq w \leq 1$) and recovery with time scale, τ_{syn}. For $GABA_A$ inhibition this time scale can be 3-10 ms. Would a G_{syn}-based I_{syn} allow for the dynamic effects and brief intra-SWR events that you find for pulse-coupling in the asymptotic frequency vs I_E. How would the dynamics of IFA be altered? Reference to the earlier paper (Donovo et al., 2018) and synaptic filtering is barely adequate. I would like to see also a simulation of the authors' LIF network with $g_{syn}(t) = G_{syn}\, w(t)$ for your typical ramping $I_E(t)$ with, say, two illustrative values for τ_{syn} and G_{syn}. It could be in Supp Info. It's surprising to us why decreasing G_{syn} (GABA blockers) in Donoso et al. (2018) did not affect the network frequency.

   Our simulations of your model, but with conductance-based synaptic current, suggested that there is a longer transient after a firing episode before the distribution of membrane potential becomes Gaussian-like. With large noise/small external excitatory drive, bimodal or trimodal distribution can exist for more than 500 ms.

3. The idea of no more than one spike per neuron per cycle is clever to avoid the resetting. Can you provide references for exploiting this feature in other studies for network firing events? Is the methodology of FPE and approximations for 'disregarding reset' generalizable to some other network cases: E-only or to E-I networks, or other architectures (say, none all-to-all)?

4. I see that you concluded from the analytic formulae in line 794 (according to Eq 41) that K needs to be sufficiently large. That's fine but I still wonder if further direct interpretation are gleaned from the formulae themselves, as they are quite complex.

   For example, do you see directly from the analysis about limits for synaptic strength? Maybe you can point to why there is a lower bound for the synaptic strength instead of an upper bound? I will accept that for μ_{max} to be large enough, the recurrent inhibition needs to be weak.

Minor Concerns:

5. I did not find a listing of individual contributions of the co-AUs to the project. I suppose you have provided this during the submission process.

6. I appreciate that you address, early on, for the naive reader, the lack of a precise definition of IFA (lines 115-124). Can you maybe say explicitly what features are *not* part of IFA? For example, some "nots" might be "network frequency decreases with I_E in the asymptotic case" (True for your network but IFA may not depend on this), and another "not" is "network frequency changes slowly (decreases/increases) with time during steady input" (There is no explicit slow time scale or adaptation in your formulation.).
   My impression is that IFA refers to the dynamic effect about deviations from steady (adiabatic) tracking of the asymptotic frequency for the instantaneous value of a dynamically changing I_E. IFA decreases with slower dynamic changes in I_E. IFA is not '*adaptation* of network frequency' for steady input. "accommodation" in IFA is specifically a term applied to responses to dynamic (e.g., ramping) stimuli as used historically to describe behavior at the individual neuron level.

7. Is it feasible under steady or slowly changing inputs (e.g. optogenetics) to obtain experimental data to support the modeling result that CV decreases with increased I_E? Is this mostly due to decreased standard deviation or increased period?
   Also from experimental data, what is the variability of local period/frequency during a SPW? Is there correlation between durations of successive SPWs?

8. I suggest that for Fig 3 the caption should explicitly describe the shaded representation of the gaussian distribution as, say, "painted on" and state explicitly that the black curves in Fig 3B are time courses of the numerical integration of the DDE, according to the equation numbers (8a, b).

9. Can you briefly describe the phenomenological 'resetting' for the DDE early on, around equations (9-10), intuitively without the math details?

10. Include horizontal lines at V_{thr}, V_{reset} for all time course plots (e.g., Fig 1A, Fig 2A, Fig 3Ai, Bi).

11. Fig 6C bottom. I think the y-axis label should be for both V and for I_E.

12. On page 3 (line 62) it's mentioned that a refractory period is ignored for simplicity. What effects are we overlooking?

13. In the middle panel of Fig 2D there's evidence of a small wiggle (gray dots) during decreasing phase of ramp. What is that about?

14. Re: the stability analysis that leads to Hopf bifurcation, HB, here and in Brunel & Hakim (1999). The authors state that the HB is supercritical at a few points in the text. It appears to be supercritical but does the analysis really distinguish that the HB is super- or sub-critical? My impression is that the determination of "direction" would require looking at higher order terms, beyond linear. Please confirm or restate.

15. Re: caption Fig 6. "Thin grey line: mean membrane potential trajectory μ(t) in response to SPW-like drive $I_E(t)$ (numerical integration of DDE Eq (8))." Please state explicitly if this integration of DDE also includes the resetting.

16. The classification of inhibition-first models into categories that are named, perturbation-based and bifurcation-based, is not meaningful enough for a general reader. Since your operating range for the DDE is for strong coupling and strong drive it is far from the HB regime of weakly modulated spiking of Brunel-Hakim; why call it bifurcation-based? You are describing distinctly large-amplitude episodic behavior. Also, what is meant by perturbation-based for the other category?

Actually, I'm wondering why you go through the HB derivation if it already is in the literature? I suppose, in your view, that an essential feature of a 'bifurcation-based' model is that the onset of oscillations occurs with weakly modulated (sparse) spiking. Increasing I_E leads to increased saturation "s" and therefore more negative μ_{min} which, in turn, overwhelms the increasing drive to produce slower network frequency. But for the Donoso et al. model (also bifurcation-based) the asymptotic frequency changes little while 's' increases to 0.8 (almost its maximum) with increasing input drive and then both 's' and frequency increase with I_E. What is the more important effect in your model of increasing drive: that frequency decreases or that 's' increases from near zero?

17. What is the initial condition for the numerical integration of the DDE, i.e. $\mu(t)$ for $-\Delta<t<0$? Since the DDE is infinite-dimensional (with respect to the initial condition), can some dynamics in Fig 8 be bistable? For example, if we initialize with the regular oscillation with I_E in the pathological oscillation/period-2 oscillation regime, will the regular oscillation be kept?

18. Along the lines of the just-previous query, what is the initial condition for the LIF network? We found in our simulation that if the membrane potentials are initialized uniformly on [V_{reset}, V_{thr}], there will be a longer initial transient with multiple clusters in the distribution of membrane potentials. Will these clusters eventually merge into one Gaussian-like cluster or is it possible that for some parameter values, there are more than one cluster (e.g., two stable clusters) as time gets large? The raster may look like ABABAB where there are two different ISIs. This may also happen when the noise level D is large.

19. Re: line 496. "wrt" should be spelled out.

20. Can you mention/summarize, say in Discussion, the features of your analytic treatment that could generalize to other applications? And highlight the advantages of the analytic approximations, beyond having the DDE derivation, over, say, just integrating the DDE.
