# Supplementary material for: Intra-ripple frequency accommodation in an inhibitory network model for hippocampal ripple oscillations
Source: PLoS Comput Biol. 2024 Feb 20;20(2):e1011886. doi: 10.1371/journal.pcbi.1011886 (PMC10923461; doi:10.1371/journal.pcbi.1011886)
Supplement: Attachment — Submitted filename: response_to_reviewer_reports.pdf [file pcbi.1011886.s005.pdf]

Responses to reviewer reports

for PLOS CB on "Intra-ripple frequency accommodation in an inhibitory network model for hippocampal ripple oscillations"

by Natalie Schieferstein, Tilo Schwalger, Benjamin Lindner, Richard Kempter

Data Availability Statement

All code written in support of this publication is publicly available at `https://github.com/NatalieSchieferstein/interneuron_ripples_with_ifa.git`. We have used Zenodo to assign a DOI to the repository: <DOI will be made available after acceptance>. An exemplary simulation input file is available from `https://zenodo.org/doi/10.5281/zenodo.10018496`.

1 Reviewer 1

In this paper, Schieferstein et al., look at the theoretical mechanisms of intra-frequency accommodation (IFA) of hippocampal ripple oscillations (>150 Hz) in a fully connected network of N inhibitory cells. Using the integrate-and-fire passive model, they develop an analytical mean-field approach for the large N condition to derive a drift-based approximation. This approximation is used to explain how IFA continuously emerges in response to symmetrical fast input ramps accompanying sharp-waves. They elegantly show that under these conditions, a hysteresis emerges in the trajectory of membrane potential from the minimal level to the spike threshold that controls cycle duration in response to fast symmetric changing drive. The paper is well written, and in spite of the strong theoretical background, it is easy to read. Results are clearly presented and contribute to a better understanding of the behavior of inhibition-first models of ripple oscillations developed by the senior author's previous work. There is value in adopting an analytical solution of the model operating in a regular oscillatory regime. Apart from some specific comments, my only concern is with providing a more balanced view of the implications of results when it comes to the real biological phenomenon.

Response: We thank the reviewer for reviewing our manuscript and for the overall positive assessment.

Below comments and suggestions that I hope can be useful to improve the paper.

1. The motivation of using symmetrical inputs is justified, since the authors look to explain an asymmetric phenomenon, IFA, from the intrinsic properties of the bifurcation-based model. However, IFA emerges directly along the fast rising phase of an input after the initial transitory response, not apparently requiring the descending phase (fig.5 and 6, eq 50 for toff, and 35 for fnet). As long as the input is fast enough to drive the instantaneous cycle above the expected asymptotic frequency, IFA will emerge. While the non-trivial hysteresis effect to a symmetric input emphasizes IFA below fnet, in real case scenarios sharp-waves are not necessarily symmetric. Thus, the emphasis on the hysteresis drags the focus out to the real speed-dependent mechanism which may actually operate. The paper would benefit by better considering these aspects (the authors partially touch this

point only at the level of discussion, lines 490-495).

Response: The reviewer correctly points out that a *symmetrical* double-ramp input is not a necessary requirement for IFA. IFA can also occur for a fast upwards-only ramp. We use merely a symmetrical input in our main figures to demonstrate that the asymmetry in the instantaneous frequencies (IFA) does not require the same asymmetry in the input. In response to this reviewer comment (and Comment 2 of reviewer 2, Comment 1 of reviewer 3), we have added a new Supplementary Figure C in Appendix S2, and we have extended the discussion in section *The shape of IFA*, explaining that the hysteresis effect described by our theory occurs for various network architectures and stimulus profiles, including a ramp with long rising and short descending phase (Fig C in Appendix S2, panel d4).

2. At introduction, there are references to various computational models of ripple generation, basically, inhibition-first and excitation-first models. The authors then refer to the experimental evidence and debate, and propose that looking at transient properties such as IFA can advance model selection. Arguing that only inhibition-first models reproduces IFA, it is implicitly assumed that they are best posed to explain ripple generation. However, all this requires some corrections. First, other examples of excitation-first models, including excitatory-inhibitory networks, are proposed to explain a range of frequency behavior during ripples (e.g. PMID: 21452258, PMID: 30482688, PMID: 2112357, PMID: 21452258. By bringing the focus to inhibition-first models (based only on recurrently connected inhibitory cells) the introduction reads unbalanced. Actually, some of the cited models consider a network of excitatory and inhibitory cells to generate transient ripple oscillations (e.g., ref 32 Malerba et al. (2016)). This is important, since ripples generation requires excitatory-inhibitory interactions as experiments suggest: optogenetic activation of PV cells does not elicit ripples, while their activation by pyramidal cells does (Stark et al., 2014). Second, some of the cited references are not about computational models of inhibition-first ripple oscillations; rather they are either about up-down slow transitions, stationary gamma oscillations and/or they reflect experimental data. Finally, some of these problems drag into results and discussion (for example: line 46, line 512).

Response: Many thanks for these important notes on ripple models, excitation, and inhibition; these notes refer mainly to our Introduction and Discussion. Let us subdivide the above comment in its multiple points and answer them separately:

Point 2.1: Mixed excitatory-inhibitory ripple models. In response to this comment we have added the new section *A note on mixed excitatory-inhibitory models for ripple generation* to the Discussion, elaborating on our use of the term *model* and the classification of ripple generation models into "excitation-first" and "inhibition-first". We also point to this new section in the introduction, which we would like to keep short and focused on the motivation of the present work.

Point 2.2: Ripple generation by parvalbumin (PV) interneuron activation. We have revised the last paragraph of the Discussion, which is now headed by the title *Measurement and quantification of ripple oscillations: Population activity or local field potential?*. There we write:

> "The LFP is traditionally believed to reflect synaptic currents (Mitzdorf, 1985; Buzsáki et al., 2016), but modeling studies have suggested that action potentials of pyramidal cells can also contribute when occurring locked to a ripple rhythm

> [26, 40]. *In fact, the participation of pyramidal cells in the ripple rhythm may be crucial for the detectability of ripple power in the LFP (Stark et al., 2014).*"

We believe that the distinction between ripple power in the LFP and in the spiking activity is a potential confounding factor in the interpretation of the results of Stark et al. (2014): they indeed did not observe ripple power in the LFP in response to optogenetic activation of parvalbumin-positive (PV) interneurons. They did, however, find ripple-modulated spiking activity (Fig 6B in Stark et al., 2014), which is in line with inhibition-first models. Thus, one may need to distinguish in future experiments whether pyramidal cell activity is indeed necessary to produce ripple oscillations in the *network activity*, or whether it is merely relevant for producing a ripple signature in the *LFP* (Schomburg et al. (2012) and Ramirez-Villegas et al. (2018) provide evidence to that effect). Stark et al. (2014) themselves state that:

"PV Interneurons Can Pace Ensemble Activity at the Ripple Frequency. [...] the lack of induced LFP oscillations does not rule out a potential role of INT-INT interactions in pacing intraripple frequency of neuronal firing."

Others have successfully elicited ripples in both network activity and LFP by optogenetic activation of PV cells in CA3 *in vitro* (Schlingloff et al., 2014). We believe that this controversy is beyond the main scope of the present manuscript. However, we think that this is an important part of our Discussion (see also our Response to Point 2.4 below).

Point 2.3: Overall balance of the Introduction. We did not intend to suggest that only the bifurcation-based (now "feedback-based") inhibition-first model *can* exhibit IFA. It is merely the only model for which IFA has been studied so far, and our work focuses on explaining the mechanism in this model. We now write in the Introduction, hoping to resolve this issue:

> "... It is therefore an interesting question which of the proposed ripple models can account for IFA, and under which assumptions. So far, only the feedback-based inhibition-first ripple model has been shown to reproduce IFA in exemplary numerical simulations (Donoso et al., 2018) ..."

Also, in our Discussion (revised paragraph with new headline *IFA in other ripple models*) we provide ideas for how each of the other models may be able to account for IFA. Therefore, we believe that the paper now reads more balanced, given that we study here just one of the ripple models. An equally detailed treatment of all other models is beyond the scope of a single paper. Based on our analysis we do, however, believe that the robustness with which the bifurcation-based (now "feedback-based") model reproduces IFA sets it apart from other ripple models: IFA is virtually built-in and hard to avoid in this model under SPW-like drive.

Point 2.4: References. The reviewer requested the following references to be added to the Introduction:

- PMID: 21452258: (Taxidis et al., 2012). We apologize for this oversight (it was mentioned in the Results but overlooked in the Introduction). We now also added the reference to the Introduction and the Discussion.
- PMID: 30482688: Ramirez-Villegas et al. (2018). This reference was previously only included in the Discussion of ripples measured in LFP versus ripples measured in spiking network activity. We now added it to the Introduction, the Results (at the beginning, near the previous line 46 mentioned by the reviewer), the Methods, as well as to the new Discussion paragraph *A note on mixed excitatory-inhibitory models for ripple generation.*

- PMID: 2112357: We think that there is a typo in this ID, which points to [Naunton RF. Otitis media research: the NIH perspective. Ann Otol Rhinol Laryngol Suppl. 1990 Jun;148:12. doi: 10.1177/00034894900990s604.]

References to up-down transitions, gamma etc occured only in the first paragraph, which was intended as a more general introduction to oscillatory phenomena and their potential cognitive function, going beyond just ripple oscillations. In response to this comment we have now deleted this first paragraph. We did cite two experimental studies in the list of bifurcation-based (now "feedback-based") inhibition-first models (Buzsáki et al. (1992); Ylinen et al. (1995)), in an attempt to give credit to what we believe were the first, informal, formulations of this model idea, without an explicit computational model. We now made this distinction explicit in the first paragraph of the Results (previously line 46). We left the text around the mentioned "line 512" (now the second paragraph in section *IFA in other ripple models* of the Discussion) unchanged (*i.e.* grouping together instances of the excitation-first models with or without interneurons) based on the reasoning now detailed in the Discussion (*A note on mixed excitatory-inhibitory models for ripple generation*).

3. Fig.4 and text: it could be useful to specify that simulation data refers to the N=10000 model, where the lim to inf applies. The limit of applicability of the mean-field solution seems to draw two different scenarios above and below simulations.

   Response: We have updated the legend and caption of Fig 4 accordingly. Indeed, the sign of the error in network frequency is different at the lower compared to the upper end of the range of applicability of our theory: Our Gaussian-drift approximation first *over*estimates the network frequency, since it ignores diffusion-mediated spiking, which is especially important close to the bifurcation. Towards the point of full synchrony the network frequency is eventually *under*estimated. This type of error appears to be related to deviations of the simulated membrane potential distribution from the assumed unimodal, Gaussian shape, as it is less prominent for stronger noise and/or stronger coupling, where the Gaussian assumption is better satisfied (Fig A in Appendix S1, panel c).

4. Line 440: The experimental results of optogenetic stimulation of PV cells generating ripples is shown at CA3 in vitro, which represents a reduced experimental model (the biological complexities explaining why that experiment worked are far from the focus of the paper). The basic pyr-inh microcircuit motif was demonstrated experimentally by Bazelot & Miles in CA3 (PMID: 26728572) and confirmed in vivo by Stark in CA1 (ref 40). Importantly here, the statement "The speed-dependence of IFA is an important prediction of the bifurcation based model. . . ." may be confusing. The whole paper is about showing how the mean-field solution for the IFA can be applied far from the bifurcation regime ($I_E \gg I_E^{min}$; fig.4) and so this makes the intra-ripple accommodation phenomena not necessarily linked to the bifurcation point (which would be rather obvious) nor to inhibition-first model.

   Response: The points are well taken! Let us subdivide the above comment in its multiple points and answer them separately:

   Point 4.1: Ripple generation by parvalbumin (PV) interneuron activation (Line 440). In this line (still in the last paragraph of the Results) we highlight the most important prediction of this work, which is the speed-dependence of IFA. An even more basic prediction of any inhibition-first model is that ripple oscillations (measured in the

inhibitory population activity) can be triggered by excitatory stimulation of interneurons. We reference Schlingloff et al. (2014) as an experimental confirmation of this prediction in CA3 *in vitro*. We have elaborated in our answer to point 2.2 above how the *in vivo* CA1 study of Stark et al. (2014) has in fact also *confirmed* that the CA1 *spiking activity* becomes ripple-modulated when PV interneurons are activated optogenetically. We are aware, however, that the interpretation of the Stark et al. (2014) results regarding the role of PV interneurons in ripple generation remains controversial in the field, which is why we cautiously referenced it as "(but see (Stark et al., 2014))". We changed this to "or induce ripple-modulated spiking activity (Stark et al., 2014)".

Point 4.2: Involvement of pyramidal cells in ripple generation. We neglect pyramidal cells in our reduced model since they are not a crucial ingredient of an inhibition-first ripple model. This certainly does not imply that we question the very existence of a mixed, pyramidal-interneuron microcircuit in CA1/CA3. We have expanded our Discussion to prevent any misunderstanding in that regard (see section *Simplifying assumptions > Pyramidal cells*, and section *A note on mixed excitatory-inhibitory models for ripple generation*). The focus of Bazelot et al. (2016) was on the emergence of *sharp waves*, not ripples; nevertheless, we included this seminal work in our Introduction.

Point 4.3: Terminology: "bifurcation-based". There has been a misunderstanding of our term "bifurcation-based inhibition-first model" (see also Reviewer 2, Comment 16a), which is actually a very valuable feedback to us and inspired a change of terminology. In the original version of the manuscript, we coined two new terms: "bifurcation-based", and "perturbation-based" in order to distinguish two distinct generation mechanisms for ripples that both fall into the category of "inhibition-first". In response to the reviewer's comment we now explain the difference between these two mechanisms in more detail (see lines 25ff in the Introduction). Note that we chose the term "bifurcation-based" to hint at the essential difference between this model class and the perturbation-based model class: In "bifurcation-based" models, the ripple state is reached when the network undergoes a *bifurcation* due to increasing excitatory drive. The perturbation-based model, on the other hand, does not undergo a bifurcation. With the term "bifurcation-based" we did *not* mean to imply that all important ripple dynamics happen close to the bifurcation. In fact, as the reviewer noted correctly, we point out that IFA happens when the drive is increased far beyond the bifurcation, and our entire theory is focused on that regime. We see, however, how the term "bifurcation-based" can be misleading, and thus chose to exchange it for "feedback-based". This term points out the core difference between the two models equally well: Only when the inhibitory synaptic feedback is strong enough, a bifurcation and sustained ripple oscillations can be achieved for strong enough drive. The perturbation-based model assumes very weak (even negligible) inhibitory feedback (Malerba et al., 2016).

In conclusion: All instances of "bifurcation-based" have been replaced by "feedback-based", and an explanation of the difference between the feedback-based and perturbation-based inhibition-first ripple models can be found in the Introduction (lines 25ff) and Discussion (*IFA in other ripple models*).

5. Line 487: The statement that decreases/increases of ripple frequency are observed experimentally refers to non-physiological data, such as in ref 41 in response to treatment with GABAa receptor agonists and antagonists. This should be clarified to avoid the reader making a wrong impression.

Response: The described switch from decreasing to increasing instantaneous frequency

is found in both the physiological baseline and the drug condition of Ponomarenko et al. (2004) (their Fig. 4 A,B: all curves show an initial decrease, followed by a short increase).

6. Discussion will benefit from inserting a short paragraph on the limitations of the study/ model. Apart from the applicability range of the mean-field approximation, other aspects to be considered are: a) Real LFP signals are contributed by spatially aligned currents from pyramidal cells. This means that inhibitory potential dynamics are strongly influenced by the integration properties in post-synaptic pyramidal cells (in contrast to statement in line 525 regarding frequency structure of IPSPs), which are not considered by the model. b) only passive leaky currents are considered in eq 1, but GABAergic cells exhibit different degree of firing rate accommodation. c) short-term plasticity of inhibitory potentials are not included. Both facilitation and depression are characteristic of heterogeneous populations of basket cells. Finally, the mean-field approximation applies to any other regular oscillatory regime with different fnet (such as for gamma and ref 34 &35) and so results may not uniquely applied to the ripple phenomenon.

   Response: Many thanks for pointing us to potential weaknesses of our Discussion. In response to the reviewer's comment, we considerably expanded and deepened the Discussion, and we also added headlines. In particular, there are now extended sections called *Performance and limitations of the Gaussian-drift approximation* and *Simplifying assumptions.*

   Point 6a) We expanded the respective paragraph of the Discussion (*Measurement and quantification of ripple oscillations: Population activity or local field potential?*). What was previously line 525 has been rephrased and supported by references to previous work. Furthermore, the (possibly nonlinear) processing of inhibitory population activity by synapses and by pyramidal cells contributes (in the LFP) to higher harmonics and changes in the shape of the power spectral density but the fundamental frequency should basically remain unchanged.

   Points 6b+c) We added the paragraph *Short term plasticity and adaptation* to our Discussion of the *Simplifying Assumptions.*

   We hope that throughout the manuscript it becomes clear that it is not our goal to incorporate as much biological detail as possible, but rather, on the contrary, neglect details in order to determine the crucial ingredients for IFA and enable an analytical understanding of its mechanism. We therefore do not consider the lack of detail a limitation of this study. Simulations of more detailed models are already available in the literature. We highlighted the limitations of our analytical derivations throughout the paper, as well as in the above mentioned extended paragraph on *Performance and limitations of the Gaussian-drift approximation.* We conclude this paragraph with a brief outlook on the applicability of our theory beyond the ripple context (in response to the last sentence of the reviewer's remark above, as well as Comments 3 and 20 of Reviewer 2).

7. For the pathological fast ripple regime, other models have been proposed based on excitation-first and/or excitatory-inhibitory interactions. The paper misses the opportunity to comment this further (e.g. lines 523, 727), although not strictly necessary. Importantly, note that experimental evidence demonstrates that physiological ripples always span the 100-250Hz frequency range and anything >250 Hz is considered pathological. Given that ripple frequency in inhibitory-first models is determined by interneuron high-frequency firing rates, the mean ripple frequency in these models is not informative.

Rather, it is the dynamics of the intra-ripple adaptation what really matter. This comment is just a side note for the authors to consider if appropriate.

Response: We feel that we should take these comments into consideration. Line 523 (now in the Discussion in section *Measurement and quantification of ripple oscillations: Population activity or local field potential?*): We deleted "fast" as it seems to be misleading here (we do not want to refer to pathological fast ripples, but simply wanted to point out that ripples are fast compared to most other brain rhythms).

Line 727 (beginning of last paragraph of section *Numerical analysis of oscillation dynamics* in the Methods): This is an interesting point, but we believe it would be unjustified to make a connection to biologically observed "fast ripples" here. The pathological oscillations observed in our numerical analysis of the DDE reflect a limitation of the Gaussian-drift approximation, which breaks down towards the low-drive regime. We now emphasize this again in the Discussion (third paragraph of section *Performance and limitations of the Gaussian-drift approximation*) stating:

> "Since our approximation is purely drift based, it is not well behaved in the limit of low drive (called "pathological oscillation" in Fig 8)."

We do not claim that this reflects a potential mechanism for biologically observed fast ripples.

Finally, we would like to respond to the above statement that the "ripple frequency in inhibitory-first models is determined by interneuron high-frequency firing rates": Only in the perturbation-based inhibitory model (Malerba et al., 2016) the ripple frequency is set by the interneuron firing rate (full synchrony: all neurons fire in all cycles). The bifurcation-based (now "feedback-based") inhibitory model has the regime of sparse synchrony, where individual neurons fire sparse and stochastically, and thus the mean unit firing rate is typically much lower than the network ripple frequency (Fig. 1B and Fig. 4).

Suggestions on the paper structure and readability

8. Many equations are numbered indisctinctly in the results and method sections. For example, eq. 1 and 13 are the same, eq. 2 and 14, etc.. (though some terms are written differently). This is possibly inevitable but the reader can get lost easily and it requires attention moving all along the results and method section.

   Response: We are aware of this problem and considered showing equations just once, either in Results or in Methods. This would, however, introduce more cross references, impeding the reading flow of the Results or Methods in isolation. We thus decided to keep duplicate equations in order to keep both Results and Methods self-contained. In response to this comment (and see also Reviewer 3, Comment 15) we added a disclaimer at the very beginning of the Methods. Also all duplicate equations in the Methods are now followed by a link to the respective duplicate equation in the Results ("see Results Eq. (...)").

9. The method section is rather long, especially for the mean-field approximation sections. One the one hand this is a major part of the paper, on the other it reads very dense and to get the main point requires digging into the underlying math. A suggestion would be to provide a short summary (also considering the previous point) which integrates the figures, and moving a large part of the mathematical derivation to supplementary material (or annex).

Response: We are aware of the considerable length of the Methods. We currently do provide a short summary of the analytical approximations in the Results, in an attempt to enable the reader to follow the Results and understand the main points *without* the need to dig in the underlying math in the Methods. In the Methods, on the other hand, we aim to provide a concise, yet comprehensive explanation of our analytical derivations. We feel that splitting the content there once again, would make it harder to look up specific methodological details, since the reader wouldn't know whether to search for a piece of information in the Methods or in an additional Appendix. This is certainly a matter of taste, but we would prefer to keep all information ordered in one (Methods) section, at the expense of this section becoming long.

2 Reviewer 2

Reviewer's report for PLoS CB on
"Intra-ripple frequency accommodation in an inhibitory network model for hippocampal ripple oscillations"
by Natalie Schieferstein, Tilo Schwalger, Benjamin Lindner, Richard Kempter

2.1 Overview

This paper reports on the modeling and analysis of IFA. It provides a follow-up to an earlier paper from the Kempter lab: Donoso, Schmitz, Maier, Kempter (2018) J Neurosci The spiking model involves all-to-all coupled LIF units; coupling is by an inhibitory current pulse with propagation delay of 1.2 ms. The application is SWRs in CA1 driven by excitatory drive IE from CA3; IE is represented by a linear ramp-up then plateau then linear ramp-down with total duration of 10s ms. The goal is to provide and to analyze a MF-like approximation for the experimental and simulated observations: generally, the intra-SWR cycle frequency shows 'accommodation', decreasing throughout the stimulus but with more or less increasing frequency during the late down-ramp, depending on the speed of the down-ramp.

The treatment proceeds in a well-organized hierarchy to describe the response to increasingly detailed features of the transient input. The authors begin with strictly steady input and approximating the steady response in terms of network frequency and cell frequency vs IE (Fig 4). This analytic part alone is quite nice and of value. Then, they provide math analysis of the ramping up and ramping down in stepwise fashion, with an IE incremental step occurring at the end of each response cycle; finally they mathematically treat the linear ramp case.

The response during ramping deviates from close tracking of the asymptotic frequency, depending on the speed and direction of the changing IE . There is asymmetry (hysteresis) between increasing and decreasing IE . A substantial portion of the paper involves accounting for the deviations with simulation and math analysis; an attempt with various approximations to describe/dissect the response to transient stimuli.

The math analysis is insightful with clever approximations/assumptions, based on a Fokker-Planck description of the noisy response of the LIF network. The authors provide a valuable and novel analytic treatment for responses to a transient input. But some readers may prefer a more direct demonstration of the gaussian approximation without the detailed analysis (see our suggestion below, just before Major Concerns).

The paper is well-organized and clearly written, with generally understandable figures and captions. It's long – with Methods being longer than the main text (20 pages compared to

19 pages) and 4 pages of Supp Info. Curiously, the authors' I-only model shows decreasing asymptotic network frequency with increasing I E . This behavior contrasts with the network frequency vs IE relation seen in many cell-based models for an I-only network, including that in Donoso et al. (2018), Fig 1D, and, for example, in models for ING-type gamma oscillations the frequency typically increases with IE (Fig 12A in Wang and Buzsáki 1996; Fig 4C in Bartos, Vida and Jonas 2007).

Response: Many thanks for the concise summary of our work and the very positive assessment of the manuscript as a whole.

We incorporated the references (Wang and Buzsáki, 1996; Bartos et al., 2007) in the now extended Discussion section *The shape of the asymptotic frequency...*. In short: the ING-type gamma networks in (Wang and Buzsáki, 1996; Bartos et al., 2007) are in a state of full synchrony. It thus makes intuitive sense that the external drive, which increases the mean unit firing rate, also increases the network frequency. Our model network, on the contrary, is in a state of sparse (stochastic) synchrony, where the relation between network frequency and external drive becomes non-trivial.

The observed difference between ING-type gamma networks and our model network (a variant of the network in (Brunel and Hakim, 1999)) is also supported by previous theoretical analyses of Brunel and Hansel (2006): Both Wang and Buzsáki (1996) and Bartos et al. (2007) used the Wang-Buzsáki neuron model, which can be approximately mapped onto an exponential integrate-and-fire (EIF) model neuron (Fourcaud-Trocmé et al., 2003). The differences in oscillation dynamics in networks of LIF vs EIF neurons have been analyzed by Brunel and Hansel (2006). They distinguish two kinds of instabilities by which the network can transition from the stable asynchronous regime towards oscillations: "clustering instabilities" (neurons fire regularly in n clusters at a mean rate of f_{net}/n), and "oscillatory rate instabilities" (neurons fire stochastically at a mean rate close to f_{net}/n, where n is the index of the nearest clustering instability). Brunel and Hansel (2006) showed that EIF networks tend to enter the oscillatory regime via an $(n = 1)$-clustering-instability (*i.e.* full synchrony), unless the noise is very high, which is in line with the findings of Wang and Buzsáki (1996); Bartos et al. (2007). On the other hand, LIF networks (such as our model network, cf. Brunel and Hakim (1999)) enter the oscillatory regime via an oscillatory rate instability that emerges from an $n > 1$ clustering instability as the noise increases. Compared to EIF networks, LIF networks require less noise to move from clustering to oscillatory rate instabilities. Therefore, we often find the regime of sparse synchrony, where the network frequency is not necessarily proportional to the external drive (see also our detailed response to Major Concern 1 (b) below, and the associated new Supplementary Figure C in Appendix S2).

We offer the following suggestion before listing our major/minor concerns. The approximations, simplifying assumptions, and analysis are impressive for arriving at an analytical solution. A key factor is the gaussian profile assumption and by-passing of the cellular-threshold resetting. The DDE by itself is a valuable reduction and can be integrated numerically for demonstrating key features of IFA. Figures 3 and 4 are quite good for the case of steady input drive. Eventually, after going through much analysis in the paper, I realized that simulations of IFA for the direct numerical integration of the DDE (as in Fig 6) could be shown earlier, say, just after Fig 4. I suggest a comparison of the DDE simulations with the LIF network simulations to directly illustrate IFA (by simulation for a piecewise linear IE (t)) with time courses like those in, say, Figs 2A, 3A, 3B. Many readers may be satisfied with these demonstrations and the text could offer them the option to by-pass the results and details of the analytical approach (pages 12-16 and in Methods, pages 27-33). You could include the time course of the mean of V across cells

from the spiking network, likewise for Fig 1. The other figures could remain pretty much as they are.

Response: We thank the reviewer for this suggestion! After reading the reviewer's comment, we realized that, while for constant drive, we illustrate the dynamics of spiking network and Gaussian-drift approximation side-by-side (Fig 3), such a direct comparison is missing for the case of time-dependent drive.

After careful consideration, however, we chose not to add an additional Figure between Figs 4 and 5 (comparing IFA in spiking network and numerically integrated DDE) for the following reasons: (1) This Figure would make the paper even longer, without adding any new information: the IFA dynamics of the spiking network are fully shown in Fig. 2, and the IFA dynamics under the Gaussian-drift approximation are shown in the current Figs. 5–7. (2) Instead of emphasizing the existence of IFA in a numerical integration of the DDE for one exemplary parameter setting, we hope to convey to the reader that our paper goes far beyond that: by reducing the model's complexity we enable an *analytical* understanding of its ripple dynamics. This approach allows us to show that IFA occurs for virtually *any* parameter setting, as long as the excitatory drive changes fast enough (SPW-like).

To incorporate the Reviewer's idea of giving some readers the option to bypass details of the analytical approach, we modified the paragraph before the beginning of section *Analysis of oscillation dynamics for piecewise constant, sharp wave-like drive*. This paragraph now explains in more detail the structure of the Results around Figs. 5, 6, and 7, hopefully enabling the reader to grasp the content of the remaining Figures without reading through the mathematical derivations in detail:

> "So far we have studied the case of constant drive describing the *asymptotic* dynamics of sustained ripple oscillations observed in the long-time limit, after initial conditions have been forgotten. This will be emphasized from here on by adding a superscript "∞". The simulations in Fig 2 already suggested that IFA emerges due to a deviation of the transient dynamics from these asymptotic dynamics. We will now use the established Gaussian-drift approximation to understand why. In a first step we will maintain the assumption of constant drive and study the transient dynamics that can be induced by perturbations of the initial condition (Fig 5 A,B). With *piecewise* constant drive as a simplistic model of a SPW-like drive we then demonstrate the core mechanism of IFA: a hysteresis of the mean membrane potential dynamics (Fig 5 B,C). We then extend the same formalism to piecewise linear drive, as a more realistic approximation of SPW-like drive, and in order to demonstrate that the IFA asymmetry is preserved even when the drive is fully symmetric (Fig 6). The Gaussian-drift approximation for linear drives allows us to study the dependence of IFA on the slope of the drive. We demonstrate that the hysteresis effect causing IFA is *speed-dependent* (Fig 7) — a novel, testable prediction of the feedback-based inhibition-first ripple model matching the simulation results from the spiking network (Fig 2D). "

We also updated Figs 5, 6 and 7 with
(a) an additional panel showing the population rate $r(t)$, and
(b) a trace for the mean membrane potential $\mu(t)$,
both as predicted analytically by the Gaussian-drift approximation under time-dependent drive. We hope this facilitates the comparison with the simulation results shown in Fig 2A.

2.2 Major concerns

1. In this LIF I-only network model the synaptic current I syn is a delta-pulse, decreasing the post-synaptic V(t) by J/N=65 mV/10000. There is not a finite time course or decay time constant for Isyn . This representation leads to stronger post-synaptic hyperpolarization (decreased μ_{min}) with increased IE . The hyperpolarization can be 2-3 x 13 mV below Vrest (see Fig 2A), say to -100 mV or more negative. I urge you to make this point very explicitly in the text. Please provide intuitive explanation for this decrease of μ_{min} with increasing I_{E} . Is it that, larger I_{E} tends to speed the passage through threshold, enabling more spikes to occur in a firing episode (greater saturation, larger value of s) and thereby more inhibitory current pulses, decreased μ_{min} ? Is this the basis for decreased asymptotic frequency with I_{E}? Please give the reader an intuitive explanation for the decrease of μ_{min} with I_{E} .

   Response: We thank the reviewer for this comment, which raises two important points that had not been discussed to full extent in the initial submission: (a) The strong, periodic hyperpolarization of the membrane potentials (decrease in μ_{min}) for increasing drive I_E, and (b) the decrease in asymptotic frequency.

   (a) As the drive I_E increases, more neurons fire spikes, producing a larger population spike (note the increase in amplitude in Fig. 1A, top). Thus, the recurrent inhibitory feedback is stronger, pushing the membrane potentials to a lower μ_{min}. We added this short intuitive explanation in the paragraph immediately following the caption of Fig 4. The fact that this decrease in μ_{min} is so strong does not really depend on the absence of synaptic filters (delta pulse-coupling), but rather on the *current-based* nature of the coupling, and thus the absence of an inhibitory reversal potential. We extended our discussion (*Simplifying Assumptions > Conductance-based synapses*), highlighting this point. We furthermore point the interested reader to an extended version of the Gaussian-drift approximation in (Schieferstein, 2023) that can account for IFA in a toy model of pulse-coupled oscillators with a linear phase response curve, which mimicks the effect of an inhibitory reversal potential. For the purpose of this manuscript we decided to present the simpler case of an LIF network with current-based coupling, which can be treated fully analytically and highlights the core mechanism of IFA.

   (b) The decrease of the asymptotic frequency for increasing drive is specific to our model architecture and not a prerequisite for the emergence of IFA. This is now pointed out immediately after Fig 1 (new last paragraph of *Ripples and IFA in a spiking neural network model > Dynamics for constant drive*).

   We added a new Discussion paragraph (*The shape of the asymptotic network frequency curve as a function of external drive*) and Supplementary Figure (Fig C in Appendix S2) discussing the influence of network architecture on the shape of the asymptotic frequency and IFA.

2. With conductance-based inhibitory synaptic current there could be saturation of the synaptic activation variable, w(t), according to Isyn = Gsyn w(t) (V-Vsyn) (with modestly hyperpolarizing Vsyn and $0 \leq w \leq 1$) and recovery with time scale, τ_{syn}. For GABAA inhibition this time scale can be 3-10 ms. Would a Gsyn -based Isyn allow for the dynamic effects and brief intra-SWR events that you find for pulse-coupling in the asymptotic frequency vs I_{E} . How would the dynamics of IFA be altered? Reference to the earlier paper (Donovo et al., 2018) and synaptic filtering is barely adequate. I would like to see also a simulation of the authors' LIF network with gsyn (t) = Gsyn w(t) for

your typical ramping $I_{\mathrm{E}}(t)$ with, say, two illustrative values for τ_{syn} and Gsyn . It could be in Supp Info. It's surprising to us why decreasing G syn (GABA blockers) in Donoso et al. (2018) did not affect the network frequency. Our simulations of your model, but with conductance-based synaptic current, suggested that there is a longer transient after a firing episode before the distribution of membrane potential becomes Gaussian-like. With large noise/small external excitatory drive, bimodal or trimodal distribution can exist for more than 500 ms.

Response: In response to this comment we have (a) expanded our discussion of the differences between our reduced model and the conductance-based model of Donoso et al. (2018) (*Discussion > Simplifying assumptions*); (b) added a new Supplementary Figure C in Appendix S2 that includes (amongst other things) a direct comparison of the asymptotic oscillation dynamics and IFA in our reduced model (Figure C in Appendix S2, panel D) and in the model of Donoso et al. (2018) (Figure C in Appendix S2, panel A); (c) added a reference to the PhD thesis of Schieferstein (2023) where an extension of the Gaussian-drift approximation for a toy model of conductance-based coupling is presented (see also our response to the reviewer's Comment 1 above).

The role of pharmacology is beyond the scope of the present paper but has been discussed in (Donoso et al., 2018). Perhaps a clarifying note: Donoso et al. (2018) modelled the effect of GABA *uptake*-blockers, which essentially *in*crease the inhibitory synaptic strength as more GABA molecules remain available in the synaptic cleft for a longer time. Effectively the peak and decay time constant of inhibitory postsynaptic potentials are increased (Fig 3 of Viereckel et al. (2013)). The theory by Brunel and Wang (2003) already suggests that the ripple frequency should be only mildly affected, as it depends more strongly on the short synaptic delay and rise time constant, which are unaltered, and there is a weaker dependence on the amplitude and the decay time constant. While this analytical result only holds strictly in the low-drive regime close to oscillation onset, the simulations of Donoso et al. (2018) confirm that also at stronger drives the network frequency remains rather stable. Intuitively: as long as the network is in a state of sparse synchrony, an increase in inhibition, which lowers the mean unit firing rate, is not immediately reflected in the network frequency. Neurons still fire stochastically, jointly producing a rhythm of a similar frequency, only each neuron participates in fewer cycles.

Finally, regarding the last remark: Indeed the Gaussian shape of the membrane potential distribution is an assumption of our theory, which is never fully justified, but works well in certain parameter regimes (strong drive, strong coupling).

3. The idea of no more than one spike per neuron per cycle is clever to avoid the resetting. Can you provide references for exploiting this feature in other studies for network firing events? Is the methodology of FPE and approximations for 'disregarding reset' generalizable to some other network cases: E-only or to E-I networks, or other architectures (say, none all-to-all)?

   Response: We conclude the now extended Discussion of the *Performance and limitations of the Gaussian-drift approximation* with references to studies that used a similar first-passage-time-ansatz to compute transient firing rate output (Goedeke and Diesmann, 2008) or neglected the single-neuron reset (Badel, 2011; Chizhov and Graham, 2007, 2008; Tchumatchenko et al., 2010; Schwalger, 2021). These studies focus on uncoupled networks / the firing statistics of single units (Chizhov and Graham, 2007; Tchumatchenko et al., 2010; Badel, 2011; Schwalger, 2021) and feedforward excitatory networks (Goedeke and

Diesmann, 2008). Whether disregarding the resetting in IF networks can be used for analyzing E-only or E-I networks is an interesting question but we are not aware of any such study. In general, a simple removal of the resetting during ongoing spiking (defined by the threshold crossings of the continuous Gaussian process (Tchumatchenko et al., 2010; Badel, 2011) should work well in the fluctuation-driven regimes at low firing rates but is expected to fail in the mean-driven regime (Schwalger, 2021). Therefore, we expect that an E-only network of non-resetting neurons will behave very differently than a corresponding model with reset.

4. I see that you concluded from the analytic formulae in line 794 (according to Eq 41) that K needs to be sufficiently large. That's fine but I still wonder if further direct interpretation are gleaned from the formulae themselves, as they are quite complex. For example, do you see directly from the analysis about limits for synaptic strength? Maybe you can point to why there is a lower bound for the synaptic strength instead of an upper bound? I will accept that for $\mu_{\max}$ to be large enough, the recurrent inhibition needs to be weak.

   Response: We have rewritten Eq. (41) (now Eq. (38)) in a more intuitive way and expanded its subsequent discussion — including a derivation of an analytical approximation of the bifurcation point observed numerically in Fig 8 (transition from stable fixed point (blue) to pathological oscillation regime (red)). We added an intuitive explanation for why inhibition must be strong enough.

   There is no upper limit on the inhibitory coupling strength. The dependence of $\mu_{\max}$ on inhibitory coupling strength is weak ($\sim \sqrt{\ln(K)}$). Hence, even for strong coupling (see Fig A in Appendix S1, $K = 50$) $\mu_{\max}$ can always become "large enough" in the sense that full synchrony is reached ($\mu_{\max} > V_T + 3\sqrt{D}$). Stronger coupling just slightly increases the amount of external drive needed to reach full synchrony (see Fig B and discussion in Appendix S1).

2.3 Minor concerns

5. I did not find a listing of individual contributions of the co-AUs to the project. I suppose you have provided this during the submission process.

   Response: Yes, the author contributions have been entered in the submission system. We are reprinting them here for the reviewer's information:
   Conceptualization: NS, TS, BL, RK
   Formal Analysis: NS, TS, BL, RK
   Funding Acquisition: BL, RK
   Investigation: NS
   Methodology: NS, TS, BL, RK
   Project Administration: RK
   Software: NS
   Supervision: TS, BL, RK
   Visualization: NS
   Writing – Original Draft Preparation: NS
   Writing – Review and Editing: NS, TS, BL, RK

6. I appreciate that you address, early on, for the naive reader, the lack of a precise definition of IFA (lines 115-124). Can you maybe say explicitly what features are not part of IFA?

For example, some "nots" might be "network frequency decreases with IE in the asymptotic case" (True for your network but IFA may not depend on this), and another "not" is "network frequency changes slowly (decreases/increases) with time during steady input" (There is no explicit slow time scale or adaptation in your formulation.). My impression is that IFA refers to the dynamic effect about deviations from steady (adiabatic) tracking of the asymptotic frequency for the instantaneous value of a dynamically changing IE . IFA decreases with slower dynamic changes in I_E . IFA is not 'adaptation of network frequency' for steady input. "accommodation" in IFA is specifically a term applied to responses to dynamic (e.g., ramping) stimuli as used historically to describe behavior at the individual neuron level.

Response: We agree with the reviewer, all of the above insights are correct: In our bifurcation-based (now "feedback-based") model IFA is fundamentally a non-stationary phenomenon because the stimulus is permanently pushing the system out of the asymptotic regime. In the revised manuscript, we emphasize this dynamic nature of IFA in Sec. *The shape of IFA* of the Discussion. Note, however, that the dynamic nature of IFA is specific to the bifurcation-based (now "feedback-based") model. For example: in the bifurcation-based (now "feedback-based") model, IFA does indeed not rely on, and cannot be achieved by, an adaptation of the network frequency under constant drive (such transients are too weak to account for realistic IFA; see extended Discussion section *Modeling sharp wave-associated excitatory synaptic input to CA1 interneurons*). The perturbation-based model on the other hand, could in principle account for IFA under constant drive by assuming strong spike frequency adaptation. We added this explanation in our Discussion of *IFA in other ripple models*.

In the above mentioned introduction to IFA (right after the caption of Fig 2, formerly lines 115-124), we merely want to summarize how IFA is defined in experimentally measured ripples (based only on the spontaneous, transient ripple activity, without any knowledge of the network behavior for constant input etc); we changed this text to make the link to experiments clearer. Many of the above points cannot be mentioned at that stage, before the subsequent analysis of the network dynamics has been performed. We therefore would like to leave the introduction to IFA basically unchanged. However, some of the above mentioned "nots" are now spelled out in the expanded Discussion in the sections *The shape of the asymptotic network frequency curve..* and *The shape of IFA*.

7. Is it feasible under steady or slowly changing inputs (e.g. optogenetics) to obtain experimental data to support the modeling result that CV decreases with increased IE ? Is this mostly due to decreased standard deviation or increased period? Also from experimental data, what is the variability of local period/frequency during a SPW? Is there correlation between durations of successive SPWs?

Response: (1) It would generally be desirable to compare the asymptotic ripple dynamics under constant drive, as predicted by the bifurcation-based (now "feedback-based") model, with experimental data. There are several challenges to acquiring a biological "ground truth" for the asymptotic dynamics: Long optogenetic stimulation may induce opsin desensitization (Chater et al., 2010), and a long constant optogenetic light pulse may thus not correspond to constant photo current. If a constant excitatory stimulation of interneurons over a long time window (∼1 second) is somehow achieved, additional short term plasticity effects may occur, such as short term depression of inhibitory synapses (Kohus et al., 2016). These effects are not captured by the theory and thus impede a

direct comparison between data and theory. We emphasize in the expanded discussion of *The shape of the asymptotic network frequencies*, as well as in the newly added Supplementary Figure C in Appendix S2, that we do not give much weight to the shape of the asymptotic network frequency curve in the reduced model. If there is a biological ground truth to this curve, it likely looks different (see Figure C in Appendix S2 for how the asymptotic network frequency curve changes as the model architecture takes into account more biological detail). The decrease in CV, however, is an actual prediction of the bifurcation-based (now "feedback-based") model, which we expect to hold under various network architectures, as well as in experimental data.

(2) The decrease in CV (in the model, Fig 1B) is due to a strong decrease in the standard deviation of interspike intervals, which occurs as the regime of full synchrony is approached, where neurons fire regularly with one spike per cycle. Note that the period (mean ISI) of single neurons actually also *de*creases (Fig 1B: the mean unit firing rate increases for increasing drive). Thus the decrease in CV is certainly mediated by the decrease in ISI standard deviation, which outweighs the decreasing mean, and reflects increased spiking regularity.

(3) We are not aware of a direct quantification of the CV of interneuron ISIs during experimentally measured spontaneous ripples, and certainly nobody has quantified its dependence on the excitatory drive to interneurons. There are multiple studies reporting average interneuron firing rates during ripples, which are well below the ripple frequency range and are thus in line with the sparse synchrony regime predicted by the bifurcation-based (now "feedback-based") model (Csicsvari et al., 1999; Klausberger et al., 2003; Lapray et al., 2012; Varga et al., 2012; Stark et al., 2015); but see (Ylinen et al., 1995; Szabo et al., 2022).

(4) We are not aware of any study that has found significant correlations between the duration of successive SPWs. Kohus et al. (2016) found that the interval between two SPW-R events is correlated with the amplitude of the respective next SPW. This could be explained by a disinhibition model for sharp wave generation (Evangelista et al., 2020).

8. I suggest that for Fig 3 the caption should explicitly describe the shaded representation of the gaussian distribution as, say, "painted on" and state explicitly that the black curves in Fig 3B are time courses of the numerical integration of the DDE, according to the equation numbers (8a, b).

   Response: Thanks for spotting the missing description. We have updated the caption of Fig. 3 accordingly.

9. Can you briefly describe the phenomenological 'resetting' for the DDE early on, around equations (9-10), intuitively without the math details?

   Response: We thank the reviewer for raising an important point, the reset was indeed not explained sufficiently in the Results. We have added (a) a short intuitive introduction of the reset in the Results (after Eq (8b), lines 240f), and (b) a technical remark on the implementation of the reset mechanism in the numerical integration of the DDE in the Methods (last sentence of *Accounting for the reset mechanism*).

10. Include horizontal lines at Vthr , Vreset for all time course plots (e.g., Fig 1A, Fig 2A, Fig 3Ai, Bi).

Response: Done (see updated Figures).

11. Fig 6C bottom. I think the y-axis label should be for both V and for I E .

    Response: Yes, the y-axis applies to both the mean membrane potential and the external drive. We now explain this in the caption of Fig 5 and Fig 6. We have also updated the y-axis label to "voltage, drive" (Fig 5 A,C and Fig 6 A,C).

12. On page 3 (line 62) it's mentioned that a refractory period is ignored for simplicity. What effects are we overlooking?

    Response: Please refer to our expanded discussion of the *Simplifying assumptions > Absolute refractoriness*: As long as the refractory period does not interfere with the unimodality of the membrane potential distribution, its influence on the oscillation dynamics is negligible.

13. In the middle panel of Fig 2D there's evidence of a small wiggle (gray dots) during decreasing phase of ramp. What is that about?

    Response: The instantaneous frequencies shown in each subpanel of Fig 2D are pooled together from 50 independent simulations with the same drive. The "wiggle" seen during the ramp-down phase of the drive in the middle and right panels results from a single cycle in each simulation. This becomes clearer when simulating only the downwards ramp, starting all 50 simulations with the same initial condition (Reviewer Figure 1 and Reviewer Figure 2). Cutting off the previous history (ramp-up- and plateau-phase) reduces the jitter in oscillation phases and recovers discrete point clouds of instantaneous frequencies for each cycle (Reviewer Figure 2, left).

    The instantaneous frequencies of the third cycle (*i.e.* derived from the distance between the third and fourth population spike) produce the "wiggle" as they are an outlier from the otherwise smooth, monotonically increasing frequency trend.

    The membrane potential distribution between populations spikes 3 and 4 exhibits a bimodality resulting from the reset of the units participating in population spike 3 (Reviewer Figure 2, right). The interval between the two population spikes is too short to allow for a full return to unimodality, hence units that have participated in population spike 3 are less likely to spike in population spike 4. Such history-dependencies and effects of the non-Gaussian shape of the membrane potential distribution cannot be captured by our Gaussian-drift approximation. The wiggle phenomenon is thus outside the scope of the presented theory.

    In response to this comment we expanded our Discussion of the *Performance and limitations of the Gaussian-drift approximation*, including a short note on the wiggle phenomenon.

14. Re: the stability analysis that leads to Hopf bifurcation, HB, here and in Brunel & Hakim (1999). The authors state that the HB is supercritical at a few points in the text. It appears to be supercritical but does the analysis really distinguish that the HB is super- or sub-critical? My impression is that the determination of "direction" would require looking at higher order terms, beyond linear. Please confirm or restate.

    Response: The bifurcation is indeed supercritical, but this is not demonstrated in our

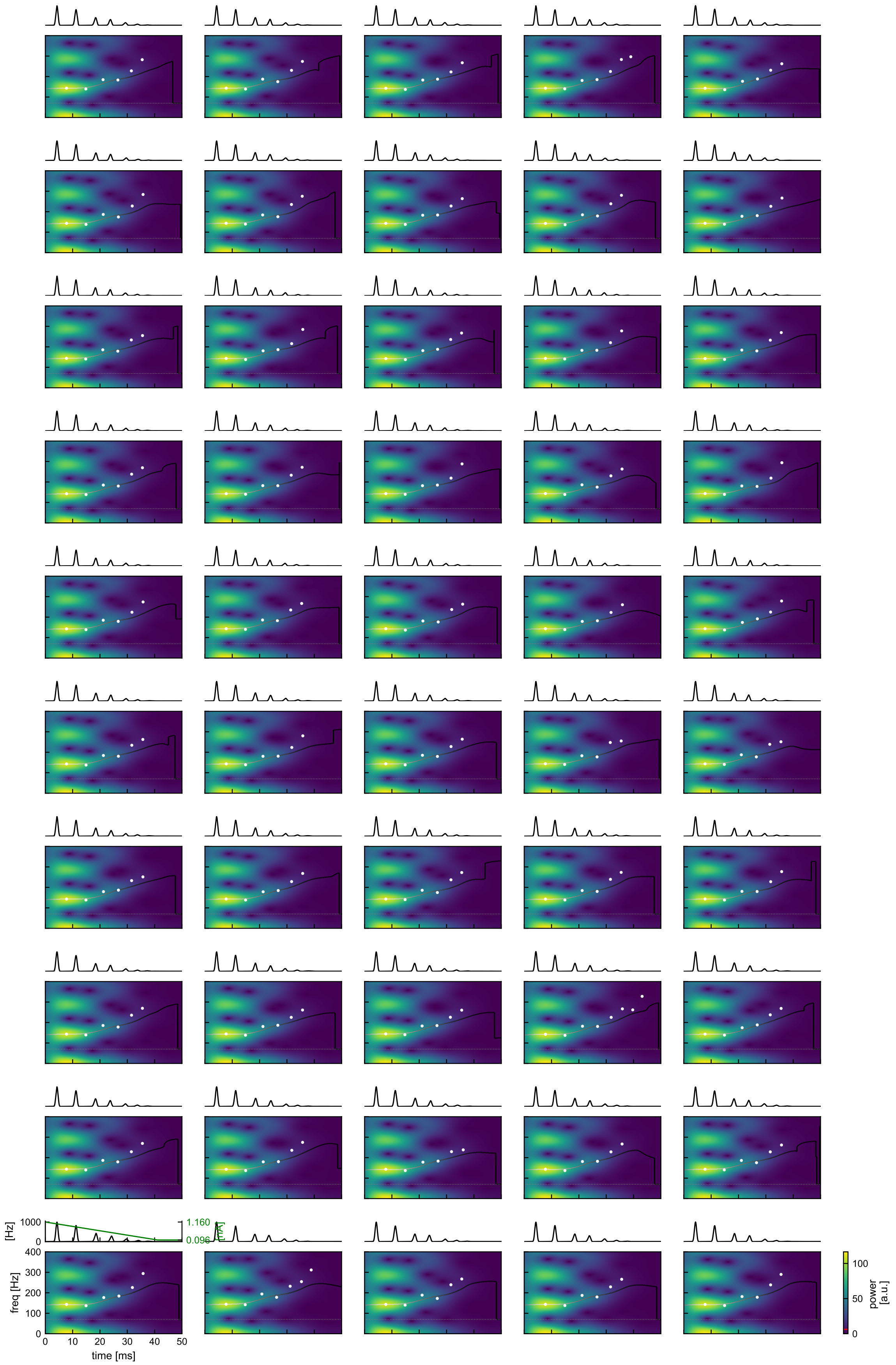

Reviewer Figure 1: Ramp-down phase of Fig 2D, middle, resimulated without previous history: All 50 simulations are shown individually. All simulations were initialized with a Gaussian membrane potential distribution centered around the asymptotic μ_{min} associated to the plateau-drive (see example in Reviewer Figure 2, right). The instantaneous frequencies shown here as white dots overlaid on the wavelet spectrograms, are pooled together in Fig 2D single plot (middle panel, gray dots during the ramping-down phase of the drive).

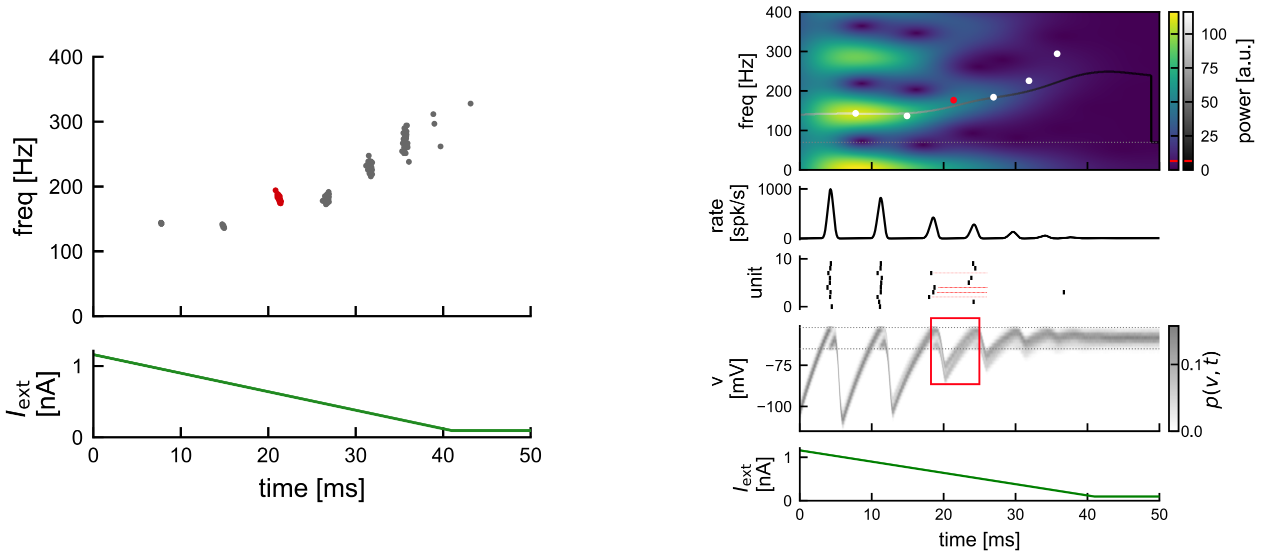

Reviewer Figure 2: Left: instantaneous frequencies from all 50 simulations shown in Reviewer Figure 1 pooled together. The "wiggle" is now clearly identifiable as a single outlier cycle (marked in red). Right: Details for one example simulation (bottom left of Reviewer Figure 1). Note that units that participated in the third population spike tend *not* to spike in the fourth population spike (red lines in raster plot), which is an indication of a residual bimodality in the membrane potential distribution from one cycle to the next (only faintly visible in voltage plot, see red square).

analysis. We cite and rely here on the result of Brunel and Hakim (1999) who indeed considered higher (up to third) order terms in a "weakly non-linear analysis" to derive the Hopf normal form and show that the bifurcation is supercritical. We assume that the following sentence (immediatly after the Caption to Fig 1) may have been confusing:

> "In the mean-field limit $N \to \infty$, the transition to this oscillatory state is sharp (a supercritical Hopf bifurcation) and *the transition point* can be determined by a linear stability analysis of the stationary state (Brunel and Hakim, 1999)."

We addded "*the transition point*" in order to clarify: The supercritical nature of the Hopf bifurcation can only be determined in a weakly nonlinear analysis considering third order terms. The location of the bifurcation, however, can be approximated in a linear stability analysis. Both analyses have been performed by Brunel and Hakim (1999).

15. Re: caption Fig 6. "Thin grey line: mean membrane potential trajectory $\mu(t)$ in response to SPW-like drive $I_{\mathrm{E}}(t)$ (numerical integration of DDE Eq (8))." Please state explicitly if this integration of DDE also includes the resetting.

    Response: We thank the reviewer for raising this point. The distinction between numerical and analytical quantities, as well as the incorporation of the rest, were indeed not clear enough. The reset mechanism is applied *throughout the paper* (only Methods Fig. 9 compares dynamics with and without reset). We hope that this is clarified now that we introduce the reset more explicitly in the second paragraph after Eq. (8b) of the Results (this was done also in response to the reviewer's Comment 9).

    The reviewer's comment 15 also inspired another change: To avoid confusion between what is derived analytically or numerically, we have changed Figures 5–7 such that *all*

quantities (even the time courses of mean membrane potential and population rate, as well as the trajectories of consecutive ripple cycles under time-dependent drive) are now derived analytically (see update of Figure Captions and Methods *Analytical approximation of oscillation dynamics for linear drive > Step 2*, last paragraph). Fig 3 is now the only Results Figure showing data derived from a numerical integration of the DDE. In the caption for Fig 3Bi we added for clarity: "Gaussian-drift approximation (numerical integration of DDE (8) *with reset condition*").

16. a) The classification of inhibition-first models into categories that are named, perturbation-based and bifurcation-based, is not meaningful enough for a general reader. Since your operating range for the DDE is for strong coupling and strong drive it is far from the HB regime of weakly modulated spiking of Brunel-Hakim; why call it bifurcation-based? You are describing distinctly large-amplitude episodic behavior. Also, what is meant by perturbation-based for the other category?

    Response: We thank the reviewer for pointing out a potential source for misunderstandings! In response to this comment (see also Reviewer 1, Comment 4.3) we have (a) expanded our Introduction of the two model categories (fourth paragraph), and (b) rephrased our Discussion of the IFA dynamics in the perturbation-based model (*IFA in other ripple models*) in an attempt to provide an intuitive understanding of the model differences. Most importantly (c), we have changed our terminology from "bifurcation-based" to "feedback-based" inhibition-first ripple model, in order to prevent the potential confusion, which the reviewers pointed out correctly. Please refer to our response to Reviewer 1, Comment 4.3 for a more elaborate explanation of this change and the model differences.

    b) Actually, I'm wondering why you go through the HB derivation if it already is in the literature?

    Response: We do in fact not derive the Hopf normal form, but only cite Brunel and Hakim (1999) in that context (see also our response to the reviewer's comment 14). We did include in our Methods a short summary of the stationary solution and a linear stability analysis of the Fokker-Planck Equation for completeness and transparency, since we actually follow the notation of Lindner and Schimansky-Geier (2001) but not that of Brunel and Hakim (1999) when computing the network frequency in the bifurcation point (red markers in Fig 1). In response to this comment we moved the whole section *Stationary solution and linear stability analysis of the Fokker-Planck Equation* to the very end of the Methods and added a disclaimer highlighting that this section just reviews computations that are common and often described in the literature.

    c) I suppose, in your view, that an essential feature of a 'bifurcation-based' model is that the onset of oscillations occurs with weakly modulated (sparse) spiking. Increasing IE leads to increased saturation "s" and therefore more negative $\mu_{\min}$ which, in turn, overwhelms the increasing drive to produce slower network frequency. But for the Donoso et al. model (also bifurcation-based) the asymptotic frequency changes little while 's' increases to 0.8 (almost its maximum) with increasing input drive and then both 's' and frequency increase with I E . What is the more important effect in your model of increasing drive: that frequency decreases or that 's' increases from near zero?

Response: In the new Discussion paragraph *The shape of the asymptotic network frequency curve [...]* and in the new Supplementary Figure C in Appendix S2 we attempt to clarify that the decrease in asymptotic frequencies is in no way a crucial point of this paper or the mechanism of IFA (please also see our response to the reviewer's Comment 1). It is the increase of the saturation (transition from sparse to full synchrony) that is a hallmark of this model, which is present in other network architectures as well (Fig C in Appendix S2, a1-d1).

17. What is the initial condition for the numerical integration of the DDE, i.e. $\mu(t)$ for $-\Delta < t < 0$? Since the DDE is infinite-dimensional (with respect to the initial condition), can some dynamics in Fig 8 be bistable? For example, if we initialize with the regular oscillation with IE in the pathological oscillation/period-2 oscillation regime, will the regular oscillation be kept?

    Response: Thanks for pointing out a missing piece of information in our Methods Section! In response to this comment we have expanded our explanation of the initialization in the Methods, *Numerical analysis of oscillation dynamics*, paragraph below Eq (31). We emphasize that we have only rigorously explored "subthreshold" initial conditions with $\mu(t \leq 0) \ll V_T$.

    One could potentially perform a brute force numerical analysis to exclude the existence of a bistable regime. Instead, we would like to provide intuitive arguments for why we believe the DDE does not exhibit bistable dynamics: (a) One can easily see analytically that the DDE (Eq 30) only has one fixed point, precisely at the external drive: $\mu(t) \equiv I_{\mathrm{E}}$. If the drive (and hence location of the fixed point) is sufficiently subthreshold, the mean membrane potential can settle into it, without causing too much of a population spike (and thus inhibitory feedback): The fixed point is stable (Fig 8, blue). We now added an analytical approximation of the bifurcation point where this fixed point becomes unstable (right after Eq (38)). (b) When integrating the DDE numerically, an initial trajectory must be chosen for the mean membrane potential $\mu(t)$ during the times $t \in [-\Delta, 0]$. The initial slope $\dot{\mu}(t), t \in [-\Delta, 0]$, should be chosen consistently. There are essentially two ways to initialize the DDE:

    (1) Subthreshold: If $\mu(t \leq 0)$ is sufficiently subthreshold ($\mu(t \leq 0) + 3\sqrt{D} < V_T$), no (significant) population rate is produced and the precise initial values at times $t < 0$ are thus irrelevant for the subsequent evolution of $\mu(t)$. The same holds for subthreshold $\mu(0)$ preceded by any arbitrary past trajectory (above or below threshold) with *negative* slope ($\dot{\mu}(t < 0) < 0$) during which the population rate is clipped to zero. The examples shown in Fig 8 thus illustrate the complete range of dynamics that can be seen for subthreshold initialization.

    (2) Suprathreshold: If at any time $t \in [-\Delta, 0]$ the initial mean membrane potential is close enough to the threshold, and increasing, such that a (significant) population rate is produced, the feedback inhibition from this activity will push the trajectory of $\mu(t)$ downwards during times $t \in [0, \Delta]$. In all our simulations of such initial conditions the system subsequently either entered a subthreshold initial condition (1), or started displaying the pathological or period-2 oscillations depicted in Fig 8. In Reviewer Figure 3 we show, as suggested by the Reviewer, a numerical integration of the DDE in the pathological regime ($I_{\mathrm{E}} = 1$), using as initial condition ($t \in [-\Delta, 0]$) a snippet of the dynamics in the regular oscillation regime ($I_{\mathrm{E}} = 6.5$): As described above, the strong population rate during times $t \in [-\Delta, 0]$ induces a strong initial transient, which eventually establishes a new, "subthreshold" initial condition. The system then enters the pathological oscillations as

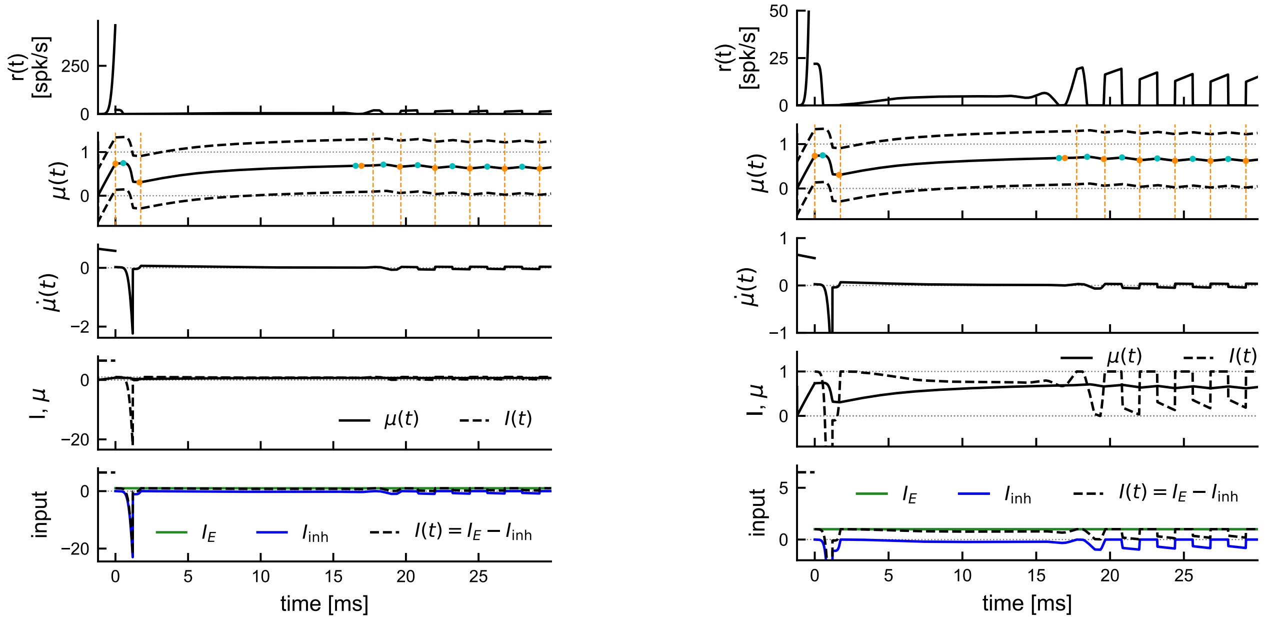

Reviewer Figure 3: Numerical integration of the DDE in the pathological regime ($I_\mathrm{E} = 1$) using as initial condition a piece of the trajectory of μ in the regular oscillation regime ($I_\mathrm{E} = 6.5$): $\mu(t) = 6.5(1 - \exp[-(t + \Delta)/\tau_m])$ for $t \in [-\Delta, 0]$. The right plot shows a zoom into the left plots. Panels from top to bottom: (1) population rate; (2) mean membrane potential $\mu(t)$; (3) slope of mean membrane potential $\dot{\mu}(t)$; (4) total current $I(t)$, compared to $\mu(t)$: intersections mark local minima and maxima of $\mu(t)$ (orange and cyan markers in (2)); (5) excitatory drive I_E (green), inhibitory feedback $I_\mathrm{I}(t)$, and summed total input $I(t)$.

shown in Fig 8. It is not possible to maintain the regular, large amplitude oscillations at a drive as low as $I_\mathrm{E} = 1$. The transients in population rate and feedback are much too weak.

18. Along the lines of the just-previous query, what is the initial condition for the LIF network? We found in our simulation that if the membrane potentials are initialized uniformly on $[V_\mathrm{reset}, V_\mathrm{thr}]$, there will be a longer initial transient with multiple clusters in the distribution of membrane potentials. Will these clusters eventually merge into one Gaussian-like cluster or is it possible that for some parameter values, there are more than one cluster (e.g., two stable clusters) as time gets large? The raster may look like ABABAB where there are two different ISIs. This may also happen when the noise level D is large.

    Response: The initial membrane potentials are drawn randomly from a uniform distribution between the reset and threshold potentials (now mentioned in Methods under Eq (16)). For our parameter settings initial transients are rather short and can be excluded from analysis by excluding the first 50 ms of the simulation (see Methods). Since our theory requires an approximately unimodal membrane potential distribution we work in a parameter regime where this is given. As we discuss in Appendix S1, cluster states are possible, especially at low noise and weak coupling strength. Such clustered activity has also been described and analyzed previously by Brunel and Hansel (2006). We are not aware of clustered activity at high noise, but we certainly have not explored all possible parameter settings across different network architectures.

19. Re: line 496. "wrt" should be spelled out.

    Response: Done.

20. Can you mention/summarize, say in Discussion, the features of your analytic treatment that could generalize to other applications? And highlight the advantages of the analytic approximations, beyond having the DDE derivation, over, say, just integrating the DDE.

    Response: We now state in the first paragraph of the Discussion that

    > "Our analytical approach presents a substantial advancement over previous, exemplary, numerical demonstrations of IFA (Donoso et al., 2018) as it allows a mechanistic understanding of the phenomenon and its parameter dependencies."

    In the last paragraph of *Performance and limitations of the Gaussian-drift approximation* we give a brief outlook on other applications:

    > "Our approach for analyzing the population rate dynamics in response to strong voltage transients may also be relevant in other contexts, such as gamma oscillations, or propagation of synchronous neural activity in synfire-chains (Diesmann et al., 1999; Memmesheimer, 2010). A hysteresis effect similar to IFA was recently observed in optogenetically evoked gamma oscillations in cat visual cortex (Lewis et al., 2021). It may be an interesting subject for future research to extend the present Gaussian-drift approximation to gamma rhythms in E-I networks. "

3 Reviewer 3

3.1 Overview

In the present MS, the authors suggest that the intra-ripple frequency adaptation observed in experiments is due to the inhibitory interneuron-based network model. In the inhibitory-based ripple model, the instantaneous frequency is inversely related to the magnitude of the instantaneous excitatory input. The basic idea of the present MS is that the instantaneous frequency depends not only on the instantaneous excitatory input, but also on the membrane potential at the end of the previous ripple cycle, endowing the system with hysteresis. Thus, when the input magnitude increases, the actual instantaneous frequency decreases more rapidly than expected, whereas when the input magnitude decreases, the instantaneous frequency increases more slowly than expected. When the input is a sufficiently short symmetric ramp, this hysteresis automatically translates to a largely monotonously decreasing instantaneous frequency.

3.2 General

I find that the MS is by and large clearly written and that the analytical and computational methods are rigorous and well explained. My main problem with the work is that the starting point for the development of the analytical model, the integrate and fire network model, yields asymptotic behavior that is inconsistent with previous theoretical and experimental findings. If the basis is inconsistent, then the entire work may be an interesting mathematical analysis but the relevance for the neurophysiology of ripples would remain unclear.

If the authors can modify the starting point for consistency with previous findings, then I think the MS warrants further consideration.

Response: We thank the reviewer for reviewing our manuscript and for the overall positive assessment of the work itself. We hope that our responses below, and the corresponding clarifications in the revised manuscript, will dispel any concerns regarding the consistency of our work with previous theoretical and experimental findings.

3.3 Major comments

1. A main inconsistency is between the present integrate-and-fire model and previous spiking and firing rate models. In the present implementation, the steady-state instantaneous frequency decreases with increased input magnitude (Fig. 1B, top; Fig. 4), whereas in the noise-driven implementation, the correlation is clearly positive (see Fig. 6 of Brunel and Hakim, 1999) or by and large flat (Fig. 2 of Brunel and Wang, 2003). It is unclear whether the opposite dependencies of frequency on input magnitude are due to (1) structural differences in the model, (2) differences in parameters, (3) different working domains, or (4) the nature of the input. This inconsistency appears for both the analytical and the spiking models. Please explain the exact differences between the present integrate and firing simulation and the Brunel and Hakim simulation, and between the present firing rate model and the 1999 one.

   Response: We will explain in the following that our model dynamics are in fact *fully consistent* with (Brunel and Hakim, 1999). Before we dive into details, let us summarize the changes we have made to the manuscript in response to this comment: We have (a) added an explanatory paragraph at the end of the Results Section *Dynamics for constant drive*; (b) expanded our discussion of *The shape of the asymptotic network frequency curve ...*; and (c) added a new Supplementary Figure (Fig C in Appendix S2) illustrating the variability of the asymptotic network frequency curve under various model architectures, ranging from the Donoso et al. (2018) model to the present variant of the Brunel and Hakim (1999) model. Figure C in Appendix S2 also shows that, independent of whether the asymptotic network frequency increases or decreases with the drive, a transient SPW-like drive elicits IFA.

   The network that we study here *is* essentially the Brunel and Hakim (1999) network (referred to above as the "noise-driven implementation"), except for one minor difference:

   The network of Brunel and Hakim (1999) is connected randomly and sparsely, while we assume full connectivity (cf. Brunel and Hansel, 2006). This does not affect the mean strength of the total inhibitory feedback, since the synaptic coupling strength is scaled down accordingly. But Brunel and Hakim (1999) thus derive a more general Fokker-Planck equation, taking into account the random *fluctuations* in inhibitory feedback (parameter H in their Eqs (3.15), (3.16)), which are not present in our network. Our network can thus be considered a special case of the Brunel and Hakim (1999) network with $H = 0$. We now state this explicitly in the first paragraph of section *Simplifying assumptions* in the Discussion.

   The findings of Brunel and Hakim (1999) (for $H = 0$) thus translate to our network, specifically: the network transitions to the oscillatory regime via a supercritical Hopf bifurcation (their Eq. (3.20), cited in our lines 100ff), and the network frequency *de*creases shortly after the bifurcation as the bifurcation parameter moves beyond the critical level

(now cited in the new Discussion section *The shape of the asymptotic network frequency curve ...*; previously line 483). Brunel and Hakim (1999) show this in the limit of short synaptic delay in their Eqs 3.21, 3.22 (details in their appendix A.2) for a more general bifurcation parameter G, which can reach the critical bifurcation level G_c by either (a) an increase in external drive (as in our manuscript), (b) an increase in inhibitory coupling strength, or (c) a *de*crease in external noise intensity.

In their Fig. 6, Brunel and Hakim (1999) show the change in network frequency with the external *noise intensity* σ_{ext} (scenario c). The external drive was fixed for the entire plot ($\mu_{\text{ext}} = 25$ mV, see Figure caption), contrary to the assumption of the Reviewer. Their Fig. 6 is thus *not* directly comparable to our Figs. 1/4 where the external drive was varied (scenario a). It still confirms, however, the analytically predicted *de*crease of the network frequency after the bifurcation: To see this, it is important to read Fig. 6 "from right to left": since the noise is used here as the bifurcation parameter (scenario c), the bifurcation from stationary to oscillatory state is crossed from right to left, as the noise intensity *de*creases. In that direction the network frequency decreases as well.

Unfortunately, Brunel and Hakim (1999) do not show the equivalent of our Fig. 1/4 in their manuscript, since they focus on the regime of weak drive around the onset of oscillations, while we explore the full range of strong drives up to the point of full synchrony. We are enclosing the Reviewer Figure 4, which shows our simulation of the Brunel and Hakim (1999) network for increasing external drive, demonstrating that the network frequency does indeed *de*crease after the bifurcation (Reviewer Figure 4B, top):

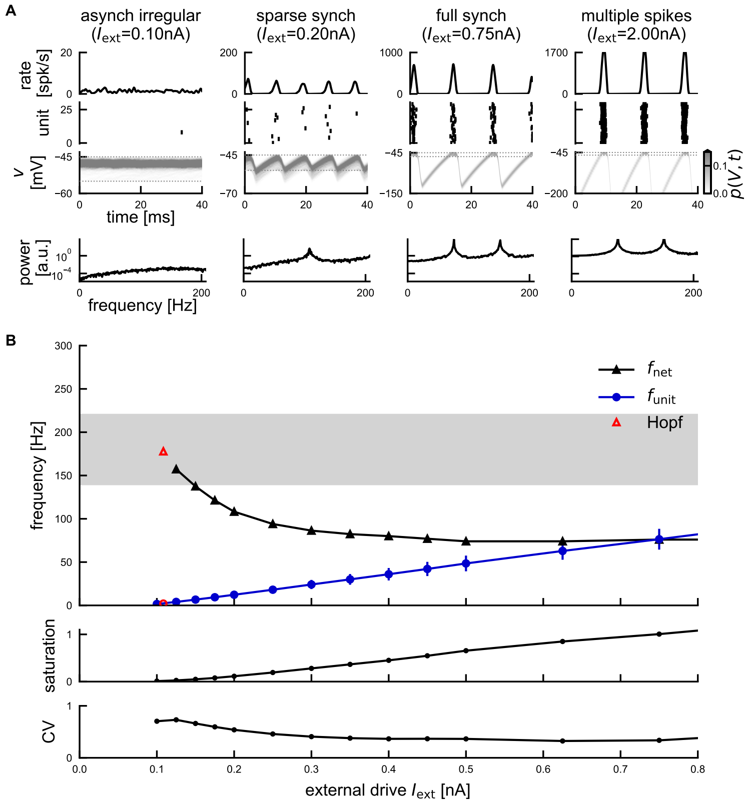

Reviewer Figure 4: In the network model of Brunel and Hakim (1999) the asymptotic network frequency also *de*creases for increasing drive . Same layout as Fig. 1 of our manuscript. Simulation of the Brunel and Hakim (1999) network under i.i.d. Gaussian white noise for increasing external drive. For comparability with our Fig 1 the drive is shown as a current (nA, see Reviewer Figure 5 for conversion to voltage as shown in Brunel and Hakim (1999)). All network parameters are set as in Figs 3-5 of Brunel and Hakim (1999). Note: The external noise intensity rises as the square root of the drive (shown in Reviewer Figure 5, left), as expected from Poisson spiking input. Red triangle: approximate Hopf bifurcation, assuming a fixed noise intensity of $\sigma_{\text{ext}} = 1.5$ mV.

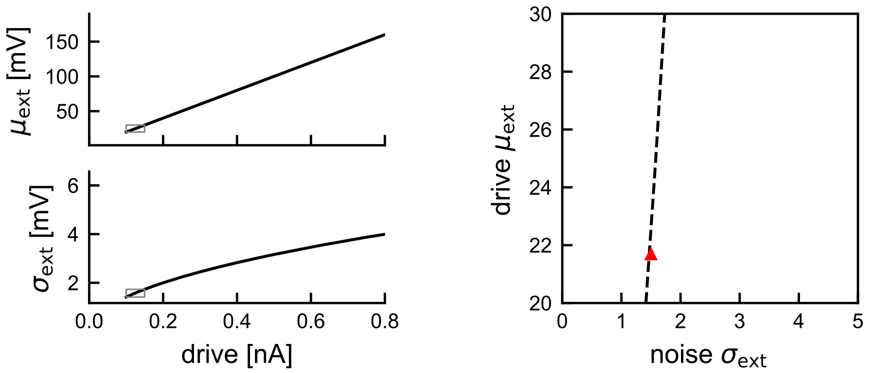

Reviewer Figure 5: The input regime studied in Brunel and Hakim (1999) relative to the range of drives shown in Reviewer Figure 4. Left: conversion of external drive from current to voltage (for an arbitrary, fixed membrane capacitance of $C = 100$ pF): $\mu_{\text{ext}} = I_{\text{ext}} \cdot \tau_m / C = 200 I_{\text{ext}}$ (mV). The external noise intensity $\sigma_{\text{ext}} \sim \sqrt{\mu_{\text{ext}}}$ increases with the mean (Poisson-like). Right: Zoom into the low-drive regime (gray squares on the left) around the bifurcation that was studied by Brunel and Hakim (1999, see their Fig. 5, right). Note that most of the dynamics illustrated in Reviewer Figure 4 (including the point of full synchrony) occurs far outside of this regime, which nicely illustrates the need for the novel Gaussian-drift approximation proposed in our manuscript.

Fig. 2B of Brunel and Wang (2003) does depict network frequency in relation to input magnitude and is also in line with our simulation results: Note that the critical input level for oscillations to emerge in this model network was ∼10 kHz, thus all network frequencies at lower inputs should be disregarded as the underlying peaks in the power spectral density are non-significant. If we focus on the network frequency for input magnitudes larger than ∼10 kHz, we see that also in this model there is a slight *de*crease in network frequency as the input magnitude rises above the critical level. Further comparison with our Fig. 1/4 is limited by the fact that (a) Brunel and Wang (2003) only show a small range of inputs that ends long before the point of full synchrony (at 30 kHz in their Fig. 2B the mean unit firing rate is still much lower than the network frequency: ∼ 50 vs. 150 Hz); (b) the network model of Brunel and Wang (2003) included *conductance-based* synapses with a double-exponential synaptic filter, which is different from the simple current-based, delta pulse coupling used in our manuscript and in (Brunel and Hakim, 1999).

In conclusion: the decrease in network frequency immediately after the bifurcation has been predicted analytically for the Brunel and Hakim (1999) network and is thus also found in our simulations. For conductance-based networks, simulations have shown that the network frequency can either decrease (Brunel and Wang, 2003; Donoso et al., 2018) or increase after the bifurcation (Geisler et al., 2005). The evolution of the network frequency far away from the bifurcation, towards the point of full synchrony, has to our knowledge only been studied numerically by Donoso et al. (2018) and in the present manuscript. Here the change in network frequency appears to depend on several aspects of the network architecture. Depending for example on the synaptic coupling and the presence of an absolute refractory period, the network frequency can either continue to decrease towards the point of full synchrony (our Fig. 1), or increase (Fig. 1D in Donoso et al., 2018). So far, we did not discuss this in depth in the manuscript since the focus of the present work is on the transient phenomenon of IFA, the mechanism of which is shown to be independent of the precise shape of the asymptotic network frequencies

under constant drive. Furthermore, there is no biological ground truth for what this curve should look like, since naturally occurring ripples are short (~ 50 ms) events with time-dependent frequency in response to time-dependent input (more on this in the response to Comment 2).

2. During spontaneous ripples, the mean ripple frequency exhibits a positive correlation with the SPW amplitude, which is consistent with the Brunel and Hakim model. Moreover, when the excitatory input is controlled experimentally using optogenetics, ripple frequency increases (see Fig. 1 of Stark et al., 2014). These inconsistencies (also see Comment 1 above) cast doubt on the relevance of the starting point of the MS (Figs. 1-4).

   Response: We thank the reviewer for this comment. As we pointed out above (Major Comment 1) there are no inconsistencies between our model and the Brunel and Hakim (1999) network: both have a decreasing asymptotic network/ripple frequency for increasing drive. Regarding the comparison of these asymptotic oscillation dynamics with experimental data we would like to make two remarks:

   (1) We do not intend to present Fig 1 as a prediction of the bifurcation-based (now "feedback-based") model that should be used for experimental verification. In fact, the shape of the asymptotic network frequency curve is quite variable depending on the details of the model network architecture. This comment (as well as Comments 1 of Reviewers 1 and 2 respectively) has inspired us to add a new Supplementary Figure C in Appendix S2, which illustrates this variability across different network architectures (Fig C in Appendix S2, a1–d1). Since our model network is quite abstract the asymptotic frequency curve might not match the one in a biologically plausible network. The focus of this work is not on a quantitative estimate of the asymptotic network frequencies under constant drive, but on understanding the ripple dynamics under time-dependent drive. To that end our theoretical approach only uses the asymptotic dynamics as a *reference point* to demonstrate that IFA emerges from a hysteresis *relative* to these dynamics: The instantaneous ripple frequency is first above, then below the asymptotic frequency. This induces an IFA-like asymmetry *independent* of the exact values of the asymptotic frequencies (see Fig C in Appendix S2, rows 2–4: The hysteresis effect under time-dependent drive is the same, independent of the shape of the asymptotic frequency curve in row i). See also the updated Discussion of *The shape of the asymptotic network frequency curve ...* and new final paragraph in Results section *Dynamics for constant drive.*

   (2) When it comes to experimental data, it is in fact not straight-forward to estimate a biological ground-truth for the dependence of the asymptotic network frequency on the external drive. In our model, the asymptotic network frequency describes the frequency of a never-ending, perfectly periodic ripple state that is reached in response to constant stimulation of the interneurons after initial transients have vanished. Such a network state is a theoretical construct and cannot be observed directly in experiments (see also our response to Comment 7 of Reviewer 2).

   Stark et al. (2014) show in their Fig 2 the ripple frequency of a spontaneous SPW-R event depending on SPW amplitude (their Fig. 2Bb), and the ripple frequency of an optogenetically induced high frequency oscillation (iHFO) depending on light intensity (their Fig. 2Cb). There are multiple difficulties in comparing these plots directly to our Fig. 1, since both horizontal and vertical axes (or x- and y-axes) do not precisely show the same quantities: The x-axis of the experimental results shows the amplitude of a *time-dependent* SPW signal measured in the local field potential (2Bb) or the intensity

of a short, optogenetic light pulse that is delivered to pyramidal cells (2Cb) — neither of which are necessarily a good proxy for a *constant* amount of excitatory *synaptic input* to *interneurons* in our model. The experimentally estimated ripple frequency (y-axes of 2Bb and 2Cb) is an average over the time-dependent instantaneous frequency of a short ($\sim$ 50ms) ripple event (spontaneous or induced). Precisely because there is the phenomenon of IFA, which may even be modulated by the slope/amplitude of the input signal as we show, it is not straight-forward to deduce the asymptotic frequency at a certain constant input level from the average instantaneous frequency response to a short input pulse around that input level.

The best experiment to probe the asymptotic network frequencies (as predicted by inhibition-first models) would likely be a long optogenetic stimulation of CA1 interneurons with varying light intensity.

3. In experiments, the frequency accommodation is clearly apparent also when the input is a constant optogenetic pulse – for long and short pulses, in CA1, DG, and neocortex. This critical feature of in vivo ripples appears to already test the predictions of the present MS but is neither accounted for nor discussed. The authors state that the "simplest form" of a SPW model is a symmetric piecewise linear ramp, but a pulse is certainly simpler from a theoretical point of view. Can the authors replicate the IFA phenomenon with a pulse?

   Response: To the best of our knowledge there is only one study to date that used optogenetic stimulation with square pulses and also quantified IFA in the induced ripples (Stark et al., 2014). Unfortunately, Stark et al. (2014) only stimulated CA1 *pyramidal* cells. Most likely a square optogenetic light pulse to pyramidal cells translates into a double-ramp-like excitatory input to the local interneurons, not unlike what we have used in this manuscript. We are not aware of any studies yet that have demonstrated IFA in response to a square pulse optogenetic activation of interneurons. Schlingloff et al. (2014) demonstrated ripples in response to optogenetic activation of parvalbumin-positive interneurons, and they also used square pulses. However, they did not quantify IFA.

   Of course it is still a valid question whether the bifurcation-based (now "feedback-based") inhibitory model exhibits IFA in response to a simple square pulse. In the Results section *Analysis of oscillation dynamics for piecewise constant, sharp wave-like drive* we discuss the transient dynamics of the model that arise under constant drive, due to perturbations in the initial conditions (a square pulse falls into this category). We emphasize that only the first cycle can have an instantaneous frequency that is different from the asymptotic frequency. A square pulse can thus *not* account for IFA. In that sense the symmetric piecewise linear ramp used in our manuscript is indeed the simplest form of drive that can account for IFA in the present model.

   In response to this comment we have now made this point more explicitly, both in the Results (lines 381) and the Discussion (*Modeling sharp wave-associated excitatory synaptic input to CA1 interneurons*).

4. The authors arrive at the hysteresis insight via an elaborate large-signal approximation to the firing rate model of Brunel and Hakim (1999), but it is not clear to me that the effort is actually warranted. The basic logic is that the latency to the spike depends on the initial membrane potential, and this can already be seen in a single neuron spiking model. Can the authors demonstrate the hysteresis effect on the membrane potential of integrate and fire neurons?

Response: We thank the reviewer for raising this concern. We would like to provide three intuitive arguments for why the present work cannot be broken down to a single-neuron model: First, we consider the trajectory of the *mean* membrane potential of an entire population of neurons, which is important since a population "spike" (i.e. a pulse with a finite width) can thus occur even if the mean membrane potential remains subthreshold. Secondly, this population spike induces a delayed negative feedback current to the same population due to the recurrent connections in the network. So yes, on most of its upstroke the mean membrane potential behaves like the membrane potential of a single integrate-and-fire neuron, relaxing exponentially towards the external drive over a time that depends trivially on the initial condition. But then the inhibitory feedback arrives, and from that moment on the total drive to "the neuron" decreases gradually in a history-dependent way, which cannot be captured in a single-neuron model. Third, there is no classic "spike-reset" mechanism of the membrane potential to a fixed voltage. Apart from a reset of the mean voltage that is scaled by the saturation s, the mean membrane potential in our model decreases due to inhibitory synaptic feedback, and its next turning point (the beginning of a new cycle) depends crucially on the strength of the delayed synaptic feedback inhibition. It is this history-dependence of the initial value of the mean membrane potential in each cycle that produces IFA in response to a double-ramp drive. In contrast, a single uncoupled integrate-and-fire neuron would not exhibit such a hysteresis when driven with a double-ramp drive. Its membrane potential would start each cycle at the same reset potential. Thus, interspike intervals (which we assume the Reviewer wants to compare here with the intervals of activity pulses of a whole population) would become shorter as the drive increases, and longer as the drive decreases, in a rather symmetrical way.

3.4 Minor comments

5. Please extend Fig. 1 to the regime where s > 1: give examples (in Fig. 1A) and extend the summary (Fig. 1B) to include the second bifurcation point.

   Response: We thank the Reviewer for this important point which made us realize that our mentioning of a "desynchronization" was misleading here: After the point of full synchrony, neurons start spiking several times per cycle, which increases the CV of ISIs, but the population rate of course still exhibits rather synchronous population spikes (now growing to pathological size). We updated the text accordingly (second paragraph below caption of Fig 1) and added an example of this regime with *multiple spikes* ($f_{\text{unit}} > f_{\text{net}}$) to Fig 1A, right. We decided, however, to keep the axis limits in Fig 1B as they were (i.e. showing only one more data point after the point of full synchrony). We feel that dedicating a large portion of the plot to the biologically unrealistic regime of $f_{\text{unit}} \gg f_{\text{net}}$ would distract the reader from the main dynamics of interest (*i.e.* between bifurcation and point of full synchrony), which we focus on in the rest of the paper.

6. Please add raster plots to Fig. 1A for every regime (e.g., 100 random spike trains out of the 10,000).

   Response: Done (see updated Fig 1).

7. Please add spectra of the membrane potentials to Fig. 1A. Do the Vm spectra also exhibit harmonics at full synchronization?

Response: The membrane potential distribution oscillates at the same frequency as the population rate (see Fig 1A). Thus, the average power spectral density of the membrane potentials (averaged across neurons) exhibits a peak at the network frequency. Since the membrane potential oscillation is not fully sinusoidal either, the power spectral density has higher harmonics as well (see Reviewer Figure 6, and our response to the reviewer's Comment 13). Since Fig 1 is already quite dense, we decided not to add an additional panel showing the power spectral densities of the membrane potentials. The oscillatory modulation of the membrane potential distribution is quite visible already in the plot of the membrane potential distributions (Fig 1A, third panel from the top), so we felt that an additional power spectral density plot would not add much.

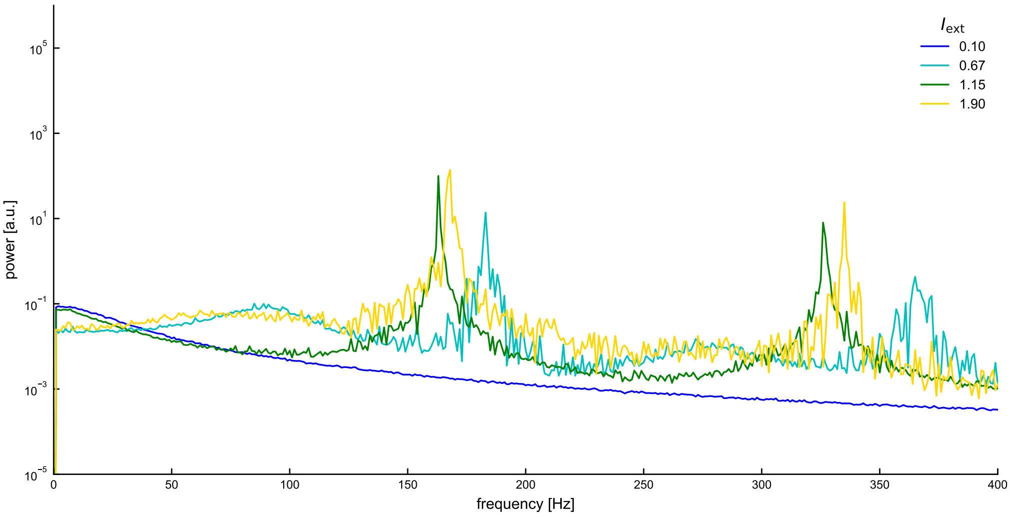

Reviewer Figure 6: Power spectral densities of membrane potential distributions under constant drive. Shown for the four example levels of drive seen in Fig 1A.

8. Please report the exact value of s at the first bifurcation, around 0.2 nA

   Response: Done (see end of first paragraph below the caption of Fig. 1).

9. Please add estimates of the dispersion of the frequency estimation to Fig. 2A, i.e., add error bars to the white dots.

   Response: Fig 2A shows one example simulation. The white dots are thus precisely the inverse of the peak-to-peak distances of the population rate shown in the second panel and have no error bars associated to them. The dispersion of frequency estimates across 50 simulations is illustrated in Fig. 2B. We decided there to simply show the instantaneous frequencies from all cycles of all simulations, since averaging and inferring errorbars would require the choice of an arbitrary bin size in the time domain, which would bias the result.

10. Please explain why the asymptotic steady state frequency dips at the end of the first ramp and the beginning of the second ramp in Fig. 2B

    Response: The asymptotic frequency curve in Fig 2B was linearly interpolated from the simulation results shown in Fig 1B (black triangles: discrete network frequency estimates for 10 different levels of drive). In Fig 2B the drive was increased up to the

approximate point of full synchrony (1.15 nA). Note that the asymptotic frequency at the next lowest level of drive in Fig 1B (∼1.03 nA) is slightly lower than the frequency at full synchrony. Thus, linear interpolation up to the point of full synchrony yields a dip with a kink.

11. In Fig. 2B, the grey dots at the plateau of the input appear to decrease in frequency. Certainly the first cluster (around -10 ms) is above the line. The resolution is not perfect, but the second cluster (around -5 ms) appears to be mostly above the line, whereas the third cluster (around 5 ms) appears to be on the line. Thus, it appears that there is some accommodation even when the input is constant – can the authors comment on this? See also Comment 3 above. If what I wrote is correct, then it seems that a main conclusion of the MS, namely that a symmetric ramp is the "minimal input" that is required to replicate the IFA phenomenon in the inhibitory network model, should be reassessed.

    Response: This observation is correct and it is what we describe in section *Analysis of oscillation dynamics for piecewise constant, sharp wave-like drive* ("Even for constant drive, there is transient dynamics if the initial mean membrane potential deviates from the asymptotic minimum $\mu^{\infty}_{\text{min}}$...") and in Fig 5. The black line in Fig 2B describes the *asymptotic* network frequency for constant input, which is reached after initial transients are forgotten, so the accommodation that the reviewer observes (at around −10 ms) is precisely this transient effect of the initial condition of the network at the onset of the plateau phase. We deliberately chose the plateau length such that the network approximately settles into the asymptotic dynamics before the drive changes again. We did not make it longer to give as much graphical space as possible to the regime of interest, i.e. the ramp-phases. Yes, strictly speaking this transient effect causes an extremely weak IFA even for constant input, but the change in frequency (from the second cycle during the plateau at around −5 ms to the third cycle at around +5 ms) is in the range of only 1 Hz (in theory: IFA only exactly one cycle, see Fig 5). So the initial transient changes in frequency in response to a sudden change of otherwise constant drive are *not* sufficient to account for biologically observed IFA (a frequency decrease by ∼10–60 Hz over multiple ripple cycles). See also our response to the reviewer's Comment 3 and the associated update of the Discussion (*Modeling sharp wave-associated excitatory synaptic input to CA1 interneurons.*

12. Please write all partial derivatives explicitly in all equations.

    Response: We checked all equations and could not find any problems in the notation of the partial derivatives (e.g. $\partial_t p(V, t)$ in Eq. 23a). All partial derivatives are explicitly written in our paper. The (regular) derivative with respect to time t has been abbreviated by a dot, e.g. in Eq. 1: $\dot{v}_i = \frac{dv_i}{dt}$ (now explained after Eq.1).

13. What is the source of the "higher harmonics" in Fig. 1A? Are these harmonics due to the fitting of integer multiple sine waves to the sequence of "delta functions", or is there rhythmicity at 400 Hz (as in Fig. 2A)?

    Response: The former: The population rate is approximately periodic but increasingly non-sinusoidal for higher levels of external drive. Thus its Fourier series indeed contains integer multiple sine waves, corresponding to the higher harmonics seen in the power spectral density. In response to this comment we extended the x-axis-limits in the lower panels of Fig 1A so that the higher harmonics also become visible for the regime of sparse

synchrony.

14. In Fig. 5A, the legend (and Fig. 5B) refers to seven examples, but only three are shown.

    Response: We slightly rephrased the caption of Fig. 5B to avoid confusion: the seven example cycles are shown fully only in B and C. Panel A only shows cycles 2 and 6, as marked in the Figure.

15. Several of the equations are repeated in both the Results and Methods sections. Please refrain from the repetition, or state explicitly next to every repeated equation which other equation it replicates (in the Methods).

    Response: We are aware of this problem and considered showing equations just once, either in Results or Methods. This would, however, introduce more cross references, impeding the reading flow of the Results or Methods in isolation. We thus decided to keep duplicate equations in order to keep both Results and Methods self-contained. In response to this comment (and see also Reviewer 1, Point 8) we added a disclaimer at the very beginning of the Methods. Also all duplicate equations in the Methods are now followed by a link to the respective duplicate equation in the Results ("see Results Eq. (..)").

Bibliography

Badel, L. (2011). Firing statistics and correlations in spiking neurons: A level-crossing approach. *Phys. Rev. E*, 84(4):041919. doi: 10.1103/PhysRevE.84.041919.

Bartos, M., Vida, I., and Jonas, P. (2007). Synaptic mechanisms of synchronized gamma oscillations in inhibitory interneuron networks. *Nat. Rev. Neurosci.*, 8(1):45–56. doi: 10.1038/nrn2044.

Bazelot, M., Teleńczuk, M. T., and Miles, R. (2016). Single CA3 pyramidal cells trigger sharp waves in vitro by exciting interneurones. *J. Physiol.*, 594(10):2565–2577. doi: 10.1113/JP271644.

Brunel, N. and Hakim, V. (1999). Fast Global Oscillations in Networks of Integrate-and-Fire Neurons with Low Firing Rates. *Neural Comput.*, 11(7):1621–1671. doi: 10.1162/089976699300016179.

Brunel, N. and Hansel, D. (2006). How Noise Affects the Synchronization Properties of Recurrent Networks of Inhibitory Neurons. *Neural Comput.*, 18(5):1066–1110. doi: 10.1162/neco.2006.18.5.1066.

Brunel, N. and Wang, X.-J. (2003). What determines the frequency of fast network oscillations with irregular neural discharges? I. Synaptic dynamics and excitation-inhibition balance. *J. Neurophysiol.*, 90(1):415–430. doi: 10.1152/jn.01095.2002.

Buzsáki, G., Anastassiou, C. A., and Koch, C. (2016). The origin of extracellular fields and currents - EEG, ECoG, LFP and spikes. *Nat. Rev. Neurosci.*, 13(6):407–420. doi: 10.1038/nrn3241.

Buzsáki, G., Horvath, Z., Urioste, R., Hetke, J., and Wise, K. (1992). High-frequency network oscillation in the hippocampus. *Science*, 256(5059):1025–1027. doi: 10.1126/science.1589772.

Chater, T. E., Henley, J. M., Brown, J. T., and Randall, A. D. (2010). Voltage- and temperature-dependent gating of heterologously expressed channelrhodopsin-2. *J. Neurosci. Methods*, 193(1):7–13. doi: 10.1016/j.jneumeth.2010.07.033.

Chizhov, A. V. and Graham, L. J. (2007). Population model of hippocampal pyramidal neurons, linking a refractory density approach to conductance-based neurons. *Phys. Rev. E*, 75(1):011924. doi: 10.1103/PhysRevE.75.011924.

Chizhov, A. V. and Graham, L. J. (2008). Efficient evaluation of neuron populations receiving colored-noise current based on a refractory density method. *Phys. Rev. E - Stat. Nonlinear, Soft Matter Phys.*, 77(1):011910. doi: 10.1103/PhysRevE.77.011910.

Csicsvari, J., Hirase, H., Czurkó, A., Mamiya, A., and Buzsáki, G. (1999). Oscillatory coupling of hippocampal pyramidal cells and interneurons in the behaving rat. *J. Neurosci.*, 19(1):274–287. doi: 10.1523/JNEUROSCI.19-01-00274.1999.

Diesmann, M., Gewaltig, M.-O., and Aertsen, A. (1999). Stable propagation of synchronous spiking in cortical neural networks. *Nature*, 402(December):529–533.

Donoso, J. R., Schmitz, D., Maier, N., and Kempter, R. (2018). Hippocampal ripple oscillations and inhibition-first network models: Frequency dynamics and response to GABA modulators. *J. Neurosci.*, 38(12):3124–3146. doi: 10.1523/JNEUROSCI.0188-17.2018.

Evangelista, R., Cano, G., Cooper, C., Schmitz, D., Maier, N., and Kempter, R. (2020). Generation of sharp wave-ripple events by disinhibition. *J. Neurosci.*, 40(41):7811–7836. doi: 10.1523/jneurosci.2174-19.2020.

Fourcaud-Trocmé, N., Hansel, D., van Vreeswijk, C., and Brunel, N. (2003). How spike generation mechanisms determine the neuronal response to fluctuating inputs. *J. Neurosci.*, 23(37):11628–11640. doi: 23/37/11628 [pii].

Geisler, C., Brunel, N., and Wang, X.-J. (2005). Contributions of intrinsic membrane dynamics to fast network oscillations with irregular neuronal discharges. *J. Neurophysiol.*, 94(6):4344–4361. doi: 10.1152/jn.00510.2004.

Goedeke, S. and Diesmann, M. (2008). The mechanism of synchronization in feed-forward neuronal networks. *New J. Phys.*, 10(1):015007. doi: 10.1088/1367-2630/10/1/015007.

Klausberger, T., Magill, P. J., Márton, L., Roberts, J., Cobden, P. M., Buzsáki, G., and Somogyi, P. (2003). Brain-state- and cell-type- specific firing of hippocampal interneurons in vivo. *Nature*, 421:844–848. doi: 10.1038/nature01374.

Kohus, Z., Káli, S., Rovira-Esteban, L., Schlingloff, D., Papp, O., Freund, T. F., Hájos, N., and Gulyás, A. I. (2016). Properties and dynamics of inhibitory synaptic communication within the CA3 microcircuits of pyramidal cells and interneurons expressing parvalbumin or cholecystokinin. *J. Physiol.*, 594(13):3745–3774. doi: 10.1113/JP272231.

Lapray, D., Lasztoczi, B., Lagler, M., Viney, T. J., Katona, L., Valenti, O., Hartwich, K., Borhegyi, Z., Somogyi, P., and Klausberger, T. (2012). Behavior-dependent specialization of identified hippocampal interneurons. *Nat. Neurosci.*, 15(9):1265–1271. doi: 10.1038/nn.3176.

Lewis, C. M., Ni, J., Wunderle, T., Jendritza, P., Lazar, A., Diester, I., and Fries, P. (2021). Cortical gamma-band resonance preferentially transmits coherent input. *Cell Rep.*, 35(5):109083. doi: 10.1016/j.celrep.2021.109083.

Lindner, B. and Schimansky-Geier, L. (2001). Transmission of noise coded versus additive signals through a neuronal ensemble. *Phys. Rev. Lett.*, 86(14):2934–2937. doi: 10.1103/PhysRevLett.86.2934.

Malerba, P., Krishnan, G. P., Fellous, J.-M., and Bazhenov, M. (2016). Hippocampal CA1 ripples as inhibitory transients. *PLOS Comput. Biol.*, 12(4):e1004880. doi: 10.1371/journal.pcbi.1004880.

Memmesheimer, R.-M. (2010). Quantitative prediction of intermittent high-frequency oscillations in neural networks with supralinear dendritic interactions. *Proc. Natl. Acad. Sci.*, 107(24):11092–11097. doi: 10.1073/pnas.0909615107.

Mitzdorf, U. (1985). Current source-density method and application in cat cerebral cortex: Investigation of evoked potentials and EEG phenomena. *Physiol. Rev.*, 65(1):37–100. doi: 10.1152/physrev.1985.65.1.37.

Ponomarenko, A. A., Korotkova, T. M., Sergeeva, O. A., and Haas, H. L. (2004). Multiple GABAA receptor subtypes regulate hippocampal ripple oscillations. *Eur. J. Neurosci.*, 20(8):2141–2148. doi: 10.1111/j.1460-9568.2004.03685.x.

Ramirez-Villegas, J. F., Willeke, K. F., Logothetis, N. K., and Besserve, M. (2018). Dissecting the synapse- and frequency-dependent network mechanisms of in vivo hippocampal sharp wave-ripples. *Neuron*, 100(5):1224–1240.e13. doi: 10.1016/j.neuron.2018.09.041.

Schieferstein, N. (2023). *Hippocampal ripple oscillations in inhibitory network models.* Phd thesis, Humboldt University Berlin.

Schlingloff, D., Kali, S., Freund, T. F., Hajos, N., and Gulyas, A. I. (2014). Mechanisms of sharp wave initiation and ripple generation. *J. Neurosci.*, 34(34):11385–11398. doi: 10.1523/JNEUROSCI.0867-14.2014.

Schomburg, E. W., Anastassiou, C. A., Buzsáki, G., and Koch, C. (2012). The spiking component of oscillatory extracellular potentials in the rat hippocampus. *J. Neurosci.*, 32(34):11798–11811. doi: 10.1523/JNEUROSCI.0656-12.2012.

Schwalger, T. (2021). Mapping input noise to escape noise in integrate-and-fire neurons: a level-crossing approach. *Biol. Cybern.*, 115(5):539–562. doi: 10.1007/s00422-021-00899-1.

Stark, E., Roux, L., Eichler, R., and Buzsáki, G. (2015). Local generation of multineuronal spike sequences in the hippocampal CA1 region. *Proc. Natl. Acad. Sci. USA*, 112(33):10521–10526. doi: 10.1073/pnas.1508785112.

Stark, E., Roux, L., Eichler, R., Senzai, Y., Royer, S., and Buzsáki, G. (2014). Pyramidal cell-interneuron interactions underlie hippocampal ripple oscillations. *Neuron*, 83(2):467–480. doi: 10.1016/j.neuron.2014.06.023.

Szabo, G. G., Farrell, J. S., Dudok, B., Hou, W.-h., Ortiz, A. L., Varga, C., Dimidschstein, J., Capogna, M., and Soltesz, I. (2022). Ripple-selective GABAergic projection cells in the hippocampus. *Neuron*, 110(12):1959–1977.e9. doi: 10.1016/j.neuron.2022.04.002.

Taxidis, J., Coombes, S., Mason, R., and Owen, M. R. (2012). Modeling sharp wave-ripple complexes through a CA3-CA1 network model with chemical synapses. *Hippocampus*, 22:995–1017. doi: 10.1002/hipo.20930.

Tchumatchenko, T., Malyshev, A., Geisel, T., Volgushev, M., and Wolf, F. (2010). Correlations and synchrony in threshold neuron models. *Phys. Rev. Lett.*, 104(5):058102. doi: 10.1103/PhysRevLett.104.058102.

Varga, C., Golshani, P., and Soltesz, I. (2012). Frequency-invariant temporal ordering of interneuronal discharges during hippocampal oscillations in awake mice. *Proc. Natl. Acad. Sci. USA*, 109(40):E2726–E2734. doi: 10.1073/pnas.1210929109.

Viereckel, T., Kostic, M., Bähner, F., Draguhn, A., and Both, M. (2013). Effects of the GABA-uptake blocker NNC-711 on spontaneous sharp wave-ripple complexes in mouse hippocampal slices. *Hippocampus*, 23(5):323–329. doi: 10.1002/hipo.22104.

Wang, X.-J. and Buzsáki, G. (1996). Gamma Oscillation by Synaptic Inhibition in a Hippocampal Interneuronal Network Model. *J. Neurosci.*, 16(20):6402–6413. doi: citeulike-article-id:134404.

Ylinen, A., Bragin, A., Nádasdy, Z., Jandó, G., Szabó, I., Sik, A., and Buzsáki, G. (1995). Sharp wave-associated high-frequency oscillation (200 Hz) in the intact hippocampus: network and intracellular mechanisms. *J. Neurosci.*, 15(1):30–46. doi: 10.1523/JNEUROSCI.15-01-00030.1995.
