# Supplementary material for: Intra-ripple frequency accommodation in an inhibitory network model for hippocampal ripple oscillations
Source: PLoS Comput Biol. 2024 Feb 20;20(2):e1011886. doi: 10.1371/journal.pcbi.1011886 (PMC10923461; doi:10.1371/journal.pcbi.1011886)
Supplement: Attachment — Submitted filename: response_to_reviewers.pdf [file pcbi.1011886.s006.pdf]

Responses to reviewer reports

for PLOS CB on "Intra-ripple frequency accommodation in an inhibitory network model for hippocampal ripple oscillations"

by Natalie Schieferstein, Tilo Schwalger, Benjamin Lindner, Richard Kempter

Reviewer 1

The authors have addressed extensively my comments and I found the new version is improved. So, I endorse publication.

Response: We thank the reviewer for the approval of our revision.

Reviewer 2

Your detailed responses to reviewers' concerns were appreciated, as were many of the modifications in your revised paper.

Response: We thank the reviewer for the approval of our revision.

Reviewer 3

The paper has been revised and improved. Two key points remain open; both were raised in my previous assessment as well as by other reviewers in the previous round. The concerns did receive detailed replies in the response letter, but insufficient clarification in the paper itself. I believe that once clarified, the paper could make an interesting contribution to the literature.

Response: We thank the reviewer for this positive assessment of our work and for carefully checking our revision.

First, the physiological observation that work aims to explain/replicate remains insufficiently clear. There are within-ripple changes in frequency, sure. But it is unclear whether it is an outcome of the system, the input to the system, etc. Furthermore, it is unclear whether there is only decrease ("adaptation") or also an increase of frequency during the beginning of the ripples (see countless wideband traces in the literature, e.g., Fig. 1 of Buzsaki et al., 1992; Fig. 2A of Csicsvari et al., 1999).

Response: Many thanks for these remarks. We think that there are three main points:

(i) There are indeed many examples of ripple oscillations of the LFP in the literature, although in most cases the within-ripple change in frequency was not quantified. The few publications in which such changes were quantified [e.g. Ponomarenko et al. (2004); Nguyen et al. (2009); Sullivan et al. (2011); Hulse et al. (2016); Stark et al. (2014); Donoso et al. (2018); Fernández-Ruiz et al. (2019)] indicate that "the instantaneous ripple frequency typically decays over the

course of a ripple event" (line 39), which does not exclude counterexamples in which the ripple frequency increases and/or is non-monotonic. We describe such cases in the Discussion (line 640ff): "Interestingly, such a switch from decreasing to increasing frequency at the end of the ripple event was also found in some experimental data [44]. Others have reported an almost monotonic decrease or even a small peak in the beginning of the event [25, 37, 43, 45]".

(ii) In our manuscript, we have associated a decrease in frequency with IFA (stated already in the Introduction, line 39). Moreover, the manuscript states that, experimentally, there is still no established definition of IFA in the field (line 136). In the Results, we state again that in the present study we assume that "A negative regression slope [over instantaneous ripple frequencies] indicates IFA." (line 147).

(iii) As the reviewer states correctly, the *mechanism* of IFA, *i.e.* "whether it is an outcome of the system, the input to the system, etc", remains unclear, which is the motivation for the present study. We show that the mechanism of IFA can differ across ripple models: In the feedback-based inhibition-first model IFA emerges intrinsically for any fast-enough external input, independent of its symmetry. In the perturbation-based model, on the other hand, the instantaneous ripple frequency is immediately proportional to the input, and thus IFA can only emerge input-driven, for an asymmetric ramp-down drive.

In summary, we think that we have (i) characterized the physiological observation that the work aims to explain, (ii) defined what we aimed to explain, and (iii) discussed various mechanisms. We therefore are unsure how the manuscript can be improved, and we believe that the concerns raised by the reviewer do not require additional changes to the manuscript.

One way to address this experimentally is to take control over the input. While I agree that light power applied to pyramidal cells does not map 1:1 to the current input to the interneurons, the two are likely to be positively correlated, in particular for weak inputs and even for recurrent networks. Looking at the spectrograms in the experimental papers once again in detail, it seems clear that when the input was constant, there was prominent frequency adaptation of the LFP frequency (e.g., Fig. 2C, Fig. 4B, and Fig. S3C of Stark et al., 2014; Fig. S4A of Fernández-Ruiz et al., 2019). It remains unclear whether the present model replicates this observation?

Second, even after adding Supplementary Figure C, it is still unclear whether the model does or does not predict frequency accommodation for a pulse input to the interneurons. In Fig. 2B and in their reply to my previous Comment 11 the authors do report frequency accommodation during fixed input. However, when addressing the previous Comment 3 the opposite is stated, while minimizing the importance of the 1 cycle caveat. This does not receive consistent and direct treatment in the manuscript either. Because the issue is key, it should not be limited to discussions during the peer review process. Rather, the behavior with respect to a pulse input should be shown clearly in a main figure and discussed heads-on in the paper.

Response: We understand the reviewer's interest in the simplest possible case, which can be summarized by the question: Can a square-pulse stimulation of interneurons induce IFA? In our manuscript we address this question from a theoretical perspective for the reduced, feedback-based inhibition-first model, and we conclude that "... stimulation with a square pulse cannot induce IFA in this model." (line 382). This is a direct consequence of our "Analysis of oscillation dynamics for piecewise constant, sharp wave-like drive" (Fig 5): The Gaussian-drift approximation predicts that under constant drive (which includes the "on"-phase of a square pulse), only the *first* oscillation cycle has an instantaneous frequency different from the asymptotic frequency, and this instantaneous frequency depends on how the initial mean membrane potential is chosen (Fig 5A). After the first cycle, the system settles back into the asymptotic dynamics immediately. We now expanded Fig 5A, adding new panels Ai/Aii, in order to

illustrate this fast convergence to the asymptotic dynamics even more clearly.

We also expanded our Discussion of *Modeling sharp wave-like drive to CA1 interneurons*, adding a separate paragraph on *Square pulse* stimulation. There we point to the Supplementary Fig C in Appendix S2, to which we added a new panel 5, showing the simulated network response to square-pulse stimulation and confirming the absence of IFA. In summary, both theory (Fig 5) and spiking network simulation (Fig C) agree in that a square pulse cannot account for IFA in the feedback-based inhibition-first model.

The experiments of Stark et al. (2014) and Fernández-Ruiz et al. (2019) do not contradict the feedback-based inhibition-first model because they did not directly test whether square-pulse stimulation of *interneurons* can induce IFA. We now made this point explicitly in the manuscript as well (fourth paragraph of section *Modeling sharp wave-like drive to CA1 interneurons > Square pulse* in the Discussion):

> "In experiments, an optogenetic square pulse stimulation of CA1 pyramidal cells has been found to elicit IFA in the local field potential (Stark et al., 2014; Fernández-Ruiz et al., 2019). An extension of the present model, including pyramidal cells and a proxy for the LFP, would be needed to determine the compatibility of the feedback-based inhibition-first model with these findings. It is plausible that a square pulse stimulation of pyramidal cells yields a non-square, double-ramp-like drive to locally connected interneurons (see the ramp-like evolution of the evoked firing rates of pyramidal cells in Fig 2E of Fernández-Ruiz et al. (2019)). As our analysis emphasizes, any such fast-changing double-ramp drive to CA1 interneurons is likely to induce IFA. For a direct square-pulse stimulation of interneurons, on the other hand, the present reduced model predicts *no* IFA in the inhibitory population activity. This prediction could be tested in future experiments."

We added the study by Fernández-Ruiz et al. (2019) to the list of references regarding IFA in the Introduction, Results, and Discussion.

Further minors changes in the manuscript, mostly correcting typos, (see red-lined version) do not alter arguments or conclusions.

Bibliography

Donoso, J. R., Schmitz, D., Maier, N., and Kempter, R. (2018). Hippocampal ripple oscillations and inhibition-first network models: Frequency dynamics and response to GABA modulators. *J. Neurosci.*, 38(12):3124–3146. doi: 10.1523/JNEUROSCI.0188-17.2018.

Fernández-Ruiz, A., Oliva, A., de Oliveira, E. F., Rocha-Almeida, F., Tingley, D., and Buzsáki, G. (2019). Long-duration hippocampal sharp wave ripples improve memory. *Science*, 364(6445):1082–1086. doi: 10.1126/science.aax0758.

Hulse, B. K., Moreaux, L. C., Lubenov, E. V., and Siapas, A. G. (2016). Membrane Potential Dynamics of CA1 Pyramidal Neurons during Hippocampal Ripples in Awake Mice. *Neuron*, 89(4):800–813. doi: 10.1016/j.neuron.2016.01.014.

Nguyen, D. P., Kloosterman, F., Barbieri, R., Brown, E. N., and Wilson, M. A. (2009). Characterizing the dynamic frequency structure of fast oscillations in the rodent hippocampus. *Front. Integr. Neurosci.*, 3. doi: 10.3389/neuro.07.011.2009.

Ponomarenko, A. A., Korotkova, T. M., Sergeeva, O. A., and Haas, H. L. (2004). Multiple GABAA receptor subtypes regulate hippocampal ripple oscillations. *Eur. J. Neurosci.*, 20(8):2141–2148. doi: 10.1111/j.1460-9568.2004.03685.x.

Stark, E., Roux, L., Eichler, R., Senzai, Y., Royer, S., and Buzsáki, G. (2014). Pyramidal cell-interneuron interactions underlie hippocampal ripple oscillations. *Neuron*, 83(2):467–480. doi: 10.1016/j.neuron.2014.06.023.

Sullivan, D., Csicsvari, J., Mizuseki, K., Montgomery, S., Diba, K., and Buzsáki, G. (2011). Relationships between hippocampal sharp waves, ripples, and fast gamma oscillation: Influence of dentate and entorhinal cortical activity. *J. Neurosci.*, 31(23):8605–8616. doi: 10.1523/JNEUROSCI.0294-11.2011.
